# Covalent capture of nitrous oxide by phosphanides

**DOI:** 10.1039/d5cc04154f

**Published:** 2025-09-12

**Authors:** Alexandre Genoux, Tak Hin Wong, Farzaneh Fadaei-Tirani, Kay Severin

**Affiliations:** a Institut des Sciences et Ingénierie Chimiques, École Polytechnique Fédérale de Lausanne (EPFL) 1015 Lausanne Switzerland kay.severin@epfl.ch

## Abstract

Potassium phosphanides with adamantyl or *tert*-butyl groups form covalent adducts with N_2_O. The adducts are sufficiently stable to permit crystallographic analyses and reactivity studies.

## Abstract 2

Potassium phosphanides with adamantly or *tert*-butyl groups form stable covalent adducts with N_2_O.

## Graphical abstract

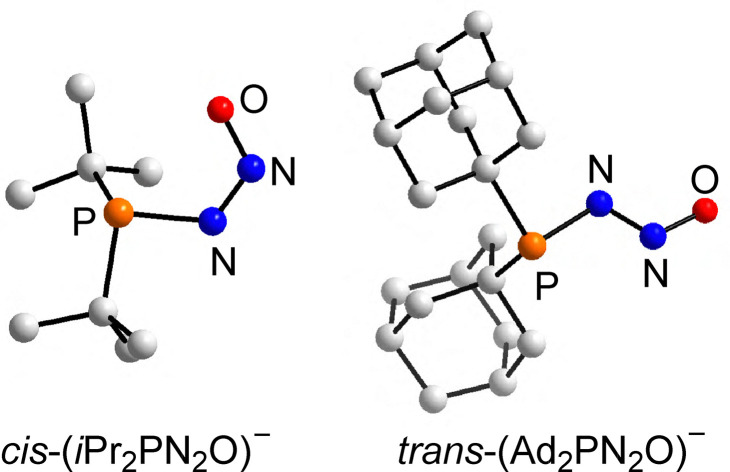

The chemical activation of nitrous oxide (N_2_O) by main group element compounds under ambient conditions is a challenging task. While potent nucleophiles can facilitate N–O bond cleavage,^1^ reports of reactions where N_2_O is captured intact are still rare.^2–6^

The capture of N_2_O with carbon-based nucleophiles can be achieved by using N-heterocyclic carbenes (Scheme 1a).^2^ The resulting diazotates have been used to prepare cationic azo dyes.^7^ The dyes are redox active^8^ and they can serve as precursors for carbene ligands.^9^

**Scheme 1:**
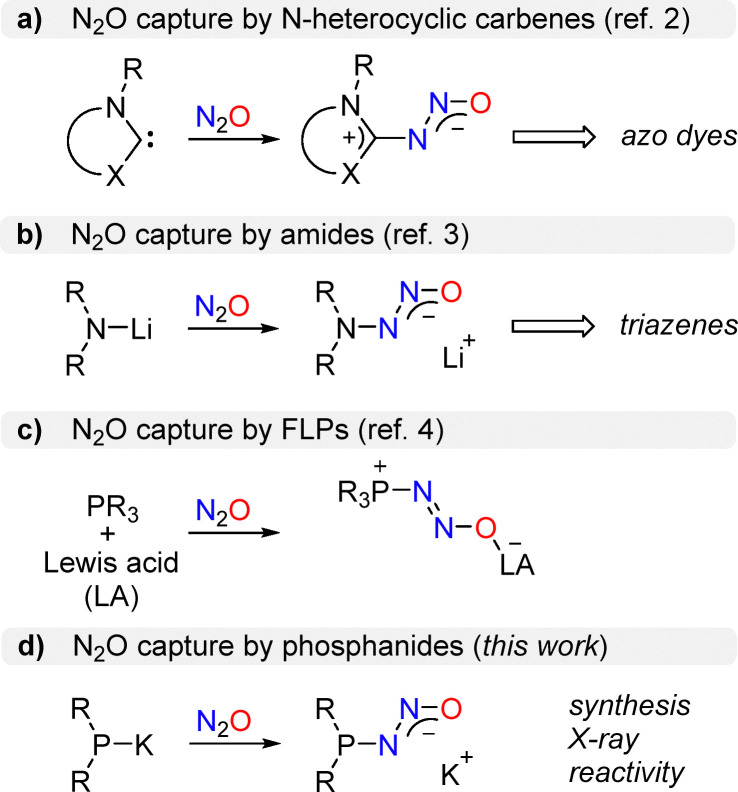
Covalent capture of nitrous oxide by N-heterocyclic carbenes (**a**), by lithium amides (**b**), or by frustrated Lewis pairs (**c**). The capture of N_2_O by potassium phosphanides is reported herein (**d**).

The capture of N_2_O *via* N–N bond formation is possible by using lithium amides (Scheme 1b).^3^ As in the case of N-heterocyclic carbenes, the reactions proceed at room temperature and under normal pressure. The products, aminodiazotates, turned out to be valuable precursors for the synthesis of triazenes.^3^ Notably, aminodiazotates have been employed for the preparation of alkynyl triazenes, which show a unique reactivity profile.^10,11^

Reactions of N_2_O with phosphorus-based compounds have predominantly resulted in oxygen atom transfer, either with^12^ or without liberation of dinitrogen.^13^ The capture of intact N_2_O can be realized by using a mixture of a phosphine (typically: P*t*Bu_3_) and a highly Lewis acidic borane or alane (Scheme 1c).^4,14^ The N_2_O adducts of these frustrated Lewis pairs (FLPs) exhibit P–N_2_O–B or P–N_2_O–Al linkages.

Herein, we demonstrate that bulky dialkylphosphanides are capable of capturing N_2_O to form phosphinodiazotates, a new class of covalent N_2_O adducts (Scheme 1d). The synthesis, structural characterization, and reactivity of these adducts are presented below.

First, we investigated the reaction of Ph_2_PK with N_2_O. The addition of N_2_O to an equimolar mixture of benzyl potassium and Ph_2_PH in THF (0.1 M, RT) resulted in the formation of the dimer Ph_2_P–PPh_2_ in high yield (Scheme 2a). This type of reactivity is characteristic of diphenylphosphanides.^15^ Moreover, it is reminiscent of the reaction between PhLi and N_2_O, in which biphenyl is one of the main products.^16^

**Scheme 2:**
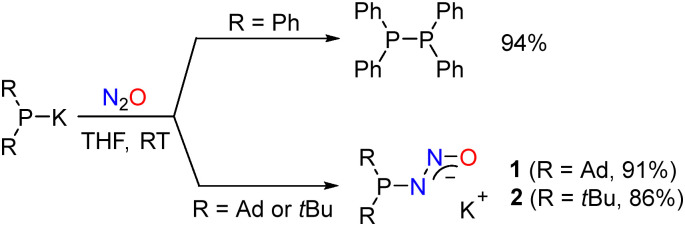
Reactions of potassium phosphanides with N_2_O, resulting in the formation of the Ph_2_PPPh_2_ or the diazotates 1 and 2.

A distinct behavior was encountered when using dialkylphosphanides with bulky adamantyl or *tert*-butyl substituents. When an equimolar mixture of benzyl potassium and Ad_2_PH in THF (0.1 M) was subjected to an atmosphere of N_2_O, the potassium phosphinodiazotate 1 was obtained in the form of a white precipitate in high yield (Scheme 2), with a detectable amount of the side product diadamantylphosphine oxide (<10%). Similarly, (*t*Bu)_2_PK reacted with N_2_O to give the corresponding potassium diazotate 2.

The diazotates of 1 and 2 were poorly soluble in THF. An increased solubility could be achieved by adding [2.2.2]cryptand, facilitating a solution-based analysis and crystallization attempts. Single crystals were obtained by layering hexane onto solutions of 1 or 2 and [2.2.2]cryptand in THF at −50 °C. Crystallographic analyses corroborated the presence of phosphinodiazotates (Fig. 1).

**Fig. 1:**
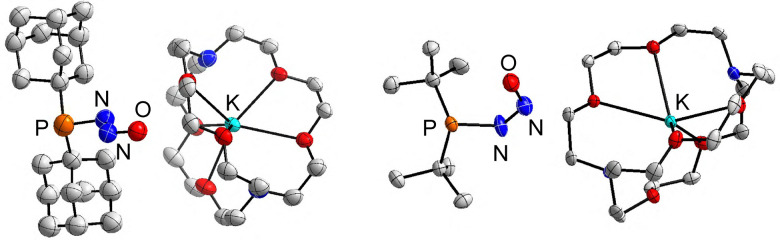
Molecular structure of the [2.2.2]cryptand complexes of 1 and 2, as determined by single-crystal X-ray analysis. Hydrogen atoms are not shown for clarity. The thermal ellipsoids are at 40% probability. Selected bond lengths (Å) and angles (°): 1: P–N 1.723(7), N–N 1.257(9), N–O 1.260(8), P–N–N 110.6(6), N–N–O 115.4(7); 2: P–N 1.775(3), N–N 1.274(5), N–O 1.276(5), P–N–N 118.3(3), N–N–O 118.4(4).

The N_2_O group in the anionic (Ad_2_PN_2_O)^−^ adopts a bent geometry. The Ad_2_P group is positioned *trans* to oxygen, with bent angles of P–N–N = 110.6(6)° and N–N–O = 115.4(7)°. The length of the N–N bond, 1.257(9) Å, is similar to that of the N–O bond (1.260(8) Å). This bonding situation differs from what has been observed for the N_2_O adduct of the frustrated Lewis pair P*t*Bu_3_/B(C_6_F_5_)_3_, for which the N–N bond (1.257(2) Å) is significantly shorter than the N–O bond (1.336(2) Å), due to weakening of the N–O bond by coordination of the borane.^4*g*^ On the other hand, comparable bond lengths were observed for the P–N bond in (Ad_2_PN_2_O)^−^ (1.723(7) Å) and for the P–N bond in *t*Bu_3_P–N_2_O–B(C_6_F_5_)_3_ (1.709(1) Å). We would like to note that the quality of the diffraction data for 1 was rather poor, and the structural parameters given above should be taken with care.

The (*t*Bu_2_PN_2_O)^−^ anion in the cryptand complex of 2 adopts a different geometry, with a *cis* arrangement of the P–N–N–O unit. While uncommon for covalent N_2_O adducts, such a geometry has been observed for the N_2_O adducts of some N-heterocyclic carbenes^2*a*,*e*^ and for aminodiazotates.^3^ The N–N the N–O bonds in (*t*Bu_2_PN_2_O)^−^ have again very similar lengths, with values of 1.274(5) Å and 1.276(5) Å, respectively.

Additional insight into the bonding in (Ad_2_PN_2_O)^−^ and (*t*Bu_2_PN_2_O)^−^ was obtained from DFT calculations at the M062X/Def2-TZVPP level of theory (for details, see the SI, Section 6). The optimized geometries of 1_calc_ and 2_calc_ were in good agreement with the crystallographically determined structure. As evidenced by the energy plots (see Fig. S30 for 1 and Fig. S34 for 2) the *cis* and *trans* isomers for both phosphinodiazotates are very close in energy (<1 kcal mol^−1^). A natural population analysis revealed a higher charge density at the N atoms adjacent to P when compared to that of the terminal O atoms. A similar situation was found for the N_2_O adducts of N-heterocyclic carbenes.^2*d*^ The Wiberg bond indices show partial double bond character for the N–N bonds and the N–O bonds. Overall, the computational results corroborate the strong electron delocalization of the diazotate group.

The reactivity of the phosphinodiazotates 1 was examined in a series of experiments. Upon addition of degassed water, the corresponding phosphine oxide was formed along with the liberation of N_2_. In the absence of moisture and oxygen, solutions of 1 are stable at room temperature for days. At elevated temperatures (60 °C in DMF), 1 was found to decompose into the corresponding phosphine oxide (Ad)_2_P(O)H. This type of reactivity is in line with what has been reported for FLP/N_2_O adducts.^4^

The addition of triethylborane (1 equiv.) to a suspension of 1 in THF at room temperature gave a homogeneous, colorless solution. *In situ*^31^P NMR analyses of the reaction mixtures indicated the complete conversion of the diazotate into a defined new compound (3) in less than 10 min (Scheme 3). A compound with a similar ^31^P NMR signal as 3 was obtained when 1 was combined with BPh_3_ instead of BEt_3_ (*δ*_P_ = 84.2 and 87.6 ppm for 3 and 4, respectively).

**Scheme 3:**
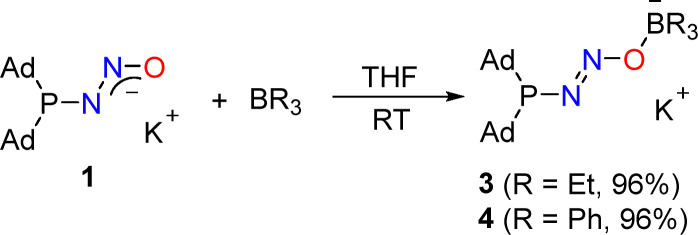
Synthesis of the borane adducts 3 and 4.

Single-crystal X-ray diffraction analysis of 3 showed that the borane had bound to the terminal oxygen atom of the diazotate group (Fig. 2, top). The P–N–N–O–B units in 3 adopt a zig-zag configuration. There are four crystallographically independent (Ad_2_PN_2_OBEt_3_)^−^ anions in the unit cell. These anions are bound *via* nitrogen and oxygen atoms to potassium ions (Fig. 2, bottom). The coordination sphere of the latter is complemented by THF molecules.

**Fig. 2:**
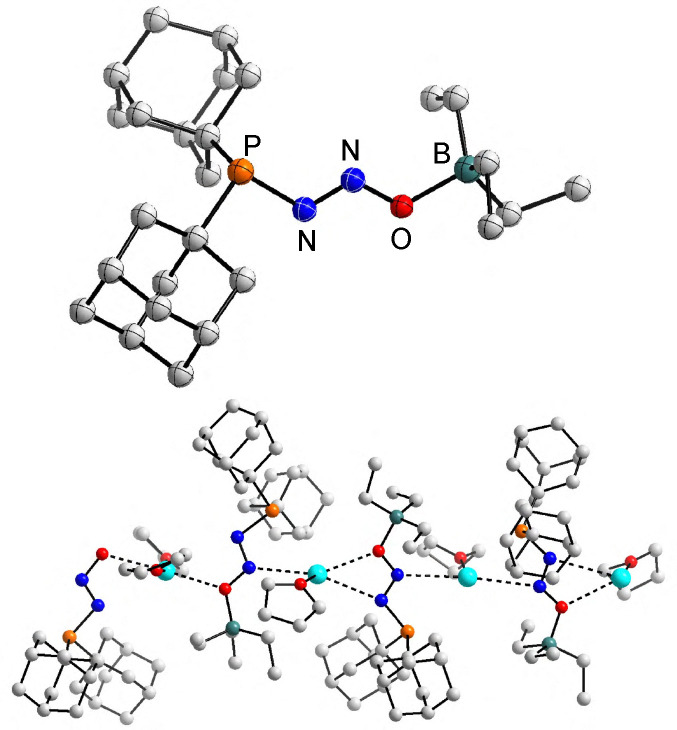
Molecular structure of one of the anions in 3, as determined by single-crystal X-ray analysis (top), along with a ball-and-stick representation showing the 1-dimensional polymeric structure of 3 (bottom). Hydrogen atoms are not shown for clarity. The thermal ellipsoids for the ORTEP representation (top) are at 40% probability.

Having established that phosphinodiazotates can form adducts with the ‘hard’ Lewis acids BEt_3_ and BPh_3_, we turned our attention to the ‘soft’ Lewis acid [(IPr)Au(MeCN)]BF_4_ (IPr = 1,3-bis(2,6-diisopropylphenyl)-imidazolin-2-ylidene). From a mixture of 1 and [(IPr)Au(MeCN)]BF_4_ in dichloromethane, we were able to isolate the Au^I^ complex 5 in 76% yield (Scheme 4a).

**Scheme 4:**
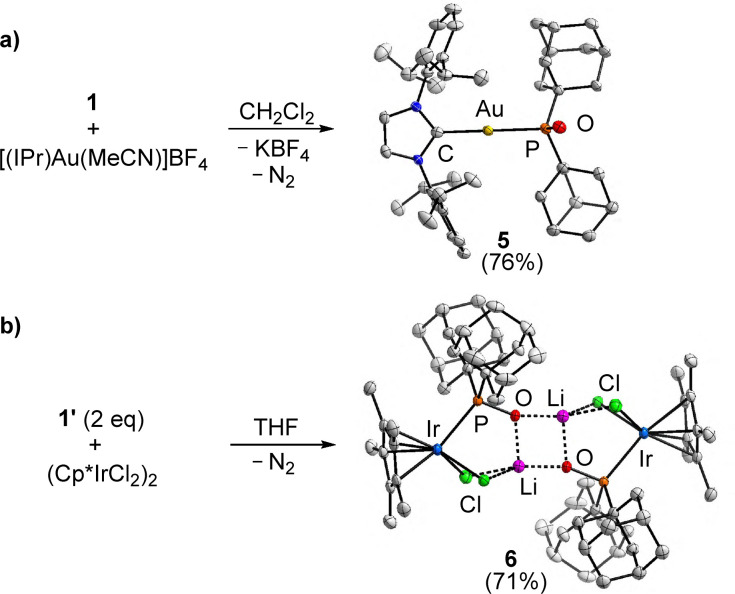
Synthesis of the complexes 5 and 6 (1′: Li^+^ instead of K^+^). The structures of the products are based on crystallographic analyses. Hydrogen atoms and co-crystallized solvent molecules are not shown for clarity. The thermal ellipsoids are at 40% probability. Selected bond lengths (Å) and angles (°): 5: Au–P 2.2972(7), Au–C 2.041(3); C–Au–P 171.26(8); 6: Ir–P 2.3841(9), Ir–Cl1 2.4413(9); Ir–Cl2 2.4270(8).

A crystallographic analysis of 5 showed that a linear Au^I^ complex with a phosphinito ligand had formed (Scheme 4a). The transformation of the phosphinodiazotate into a phosphinito required the loss of dinitrogen. Accordingly, we observed bubbles during the reaction between [(IPr)Au(MeCN)]BF_4_ and 1.

Anionic phosphinito ligands of the general formula (R_2_PO)^−^ are very strong electron donors.^17^ They have found numerous applications as ligands in transition metal catalysis.^18^ Thus far, there are only scarce reports about Au^I^ complexes with phosphinito ligands.^19^ The Au–P bond length of 2.2972(7) Å in 5 is within the expected range.^19^ NMR spectroscopic analysis in CD_2_Cl_2_ showed a single ^31^P resonance at 114.6 ppm.

Test reactions of the phosphinodiazotate 1 with other transition metal complexes such as VCl_3_(THF)_3_, PdCl_2_ and [Ru(*p*-cymene)Cl_2_]_2_ also resulted in loss of dinitrogen, as evidenced by bubble formation. For the reaction between (Cp*IrCl_2_)_2_ and the diazotate 1′ (Li^+^ instead of K^+^), we were able to isolate and crystallize a defined product, complex 6 (Scheme 4b). As in the case of 5, a phosphinito complex had formed. The negative charge of the [Cp*IrCl_2_(POAd_2_)]^−^ complex is compensated by Li^+^ cations. In the solid state, one can observe a dimer, with two Li^+^ ions being bound to the O-atoms of the phosphinito ligands and two chloro ligands (Scheme 4b).

Finally, we examined the reactivity of the diazotate 1 towards a Grignard reagent, PhMgBr. When a suspension of 1 in THF was combined with PhMgBr (3 equiv.), the P–C coupling producs Ad_2_PPh was formed in 72% yield. A similar reactivity was reported by Moss and Banger for alkyl diazotates, where they isolated products of C–C bond formation.^20^

To conclude, we have shown that potassium phosphanides with adamantly or *tert*-butyl groups form stable covalent adducts with N_2_O. These adducts represent rare examples of heteroatom-bound diazotates. Crystallographic analyses revealed distinct structures for (Ad_2_PN_2_O)^−^ (*trans* P–N–N–O) and (*t*Bu_2_PN_2_O)^−^ (*cis* P–N–N–O). The phosphinodiazotate 1 can bind intact to boranes, but reactions with transition metal complexes resulted in loss of dinitrogen, and the formation of phosphinito complexes. The use of phosphinodiazotates represents a conceptually new approach to synthesize phosphinito complexes.

A. G. and K. S. initiated the study, A. G. performed the experiments and analyzed the data, T. H. W. carried out the computational analysis, F. F.-T. collected and processed the X-ray data, and A. G. and K. S. co-wrote the manuscript. All authors discussed the results and commented on the manuscript.

This work was supported by the European Union under the Marie Skłodowska-Curie fellowship HORIZON-TMA-MSCA-PF-GF No. 1011150286, and by the Swiss National Science Foundation.

## Conflicts of interest

There are no conflicts to declare.

## Supplementary Material

CC-061-D5CC04154F-s001

CC-061-D5CC04154F-s002

## Data Availability

The data supporting this article have been included as part of the SI. Supplementary information: Containing synthetic procedures and experimental details. See DOI: https://doi.org/10.1039/d5cc04154f. CCDC 2473516 (1), 2473519 (2), 2473517 (3), 2473518 (5) and 2473515 (6) contain the supplementary crystallographic data for this paper.^21*a*–*e*^
