# Supplementary material for: Covalent capture of nitrous oxide by phosphanides
Source: Chem Commun (Camb). 2025 Sep 12;61(80):15654–7. doi: 10.1039/d5cc04154f (PMC12428274; doi:10.1039/d5cc04154f)
Supplement: CC-061-D5CC04154F-s001 [file CC-061-D5CC04154F-s001.pdf]

Supplementary Information (SI) for ChemComm.
This journal is © The Royal Society of Chemistry 2025

Electronic Supplementary Information

Covalent capture of nitrous oxide by phosphanides

Alexandre Genoux, Tak Hin Wong, Farzaneh Fadaei-Tirani and Kay Severin*

Institute of Chemical Sciences and Engineering, École Polytechnique Fédérale de Lausanne (EPFL), CH-1015 Lausanne, Switzerland

e-mail: *kay.severin@epfl.ch*

Table of Contents

	Page
1. General	S2
2. Syntheses	S3
3. Control experiments	S8
4. NMR spectra	S9
5. Single crystal X-ray analyses	S20
6. Computational details	S31
7. References	S44

1. General

Unless stated otherwise, reactions were performed under an atmosphere of dry dinitrogen or nitrous oxide (purity: 99.999%, Messer Schweiz AG) using standard Schlenk techniques or a glovebox. Solvents were purchased dry and were stored under 4 Å molecular sieves for a minimum of two days prior to use. The reagents were obtained from commercial sources and used directly in a glovebox after flushing for 10 minutes. Benzyl potassium[1] and triphenylborane[2] were prepared according to literature precedents. [(IPr)Au(MeCN)]BF_4 and [Cp*IrCl_2]$_2$ were purchased from Sigma-Aldrich (Merck).

The NMR spectra were measured on a Bruker Avance DPX-400 (^{1}H: 400 MHz) or Bruker Avance NEO-500 (^{1}H: 500 MHz). Chemical shifts are given in parts per million (ppm) relative to their solvent signals [CD_2Cl_2: 5.32 (^{1}H-NMR) and 53.84 (^{13}C-NMR); DMF-*d7*: 8.03, 2.92, 2.92 (^{1}H-NMR), 163.15, 34.89 and 29.76 (^{13}C-NMR); THF-*d8*: 1.72, 3.58 (^{1}H-NMR) 25.31 and 67.21 (^{13}C-NMR)]. Mass spectra were acquired on a LTQ Orbitrap FTMS instrument (LTQ Orbitrap Elite FTMS, Thermo Scientific) operated in the positive mode coupled with a robotic chip-based nano-ESI source (TriVersa Nanomate, Advion Biosciences). A standard data acquisition and instrument control system was utilized (Thermo Scientific), whereas the ion source was controlled by Chipsoft 8.3.1 software (Advion BioScience). Samples were loaded onto a 96-well plate within an injection volume of 5 µl. The experimental conditions for the ionization voltage were +1.4 kV and the gas pressure was set at 0.30 psi. The temperature of the ion transfer capillary was 200 °C. FTMS spectra were obtained in the 100–1000 *m*/*z* range in the reduced profile mode with a resolution set to 120,000. In all spectra, one microscan was acquired with a maximum injection time value of 1000 ms.

2. Syntheses

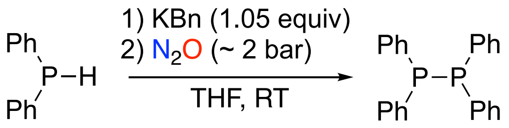

1,1,2,2-Tetraphenyldiphosphane: A dried Schlenk flask equipped with a stir bar was charged with $HPPh_2$ (372 mg, 2.00 mmol) and THF (5 mL). Benzyl potassium (273 mg, 2.10 mmol) was added at RT. After 15 min, the N_2 atmosphere was replaced by N_2O (3 vacuum/N_2O cycles) and the mixture was stirred under N_2O atmosphere (~ 2 bar) for 1 h. The solvent was then removed under reduced pressure. The crude solid was dissolved with Et_2O (5 mL) and the mixture was filtered over a fritted glass funnel. The solvent was removed under reduced pressure to give the pure product as a white solid; yield: 94% (350 mg, 0.94 mmol).

^{1}H NMR (400 MHz, CD_2Cl_2) δ 7.61 – 7.55 (m, 8H), 6.97 – 6.93 (m, 2H); **^{31}P{^{1}H} NMR** (162 MHz, CD_2Cl_2) δ –15.4. The spectroscopical data are in accordance with the reported data.[3]

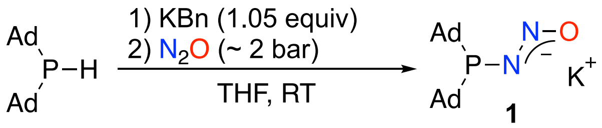

Compound 1: A dried Schlenk flask equipped with a stir bar was charged with $HPAd_2$ (302 mg, 1.00 mmol) and THF (5 mL). The mixture was cooled to –40 °C and benzyl potassium (137 mg, 1.05 mmol) was added. The mixture was allowed to warm to RT. After 15 min, the N_2 atmosphere was replaced by N_2O (3 vacuum/N_2O cycles) and the mixture was stirred under N_2O atmosphere (~ 2 bar). After 30 min, the solvent was removed under reduced pressure. The white crude was washed with cold THF (2 x 2 mL) and Et_2O (2mL) to give the product **1** as a white solid (Ad_2PN_2OK • 0.5 THF); yield: 91 % (482 mg, 0.91 mmol).

Remark: Diadamantylphosphine oxide was formed as a byproduct (~10%) alongside compound **1** and could not be separated.

Crystals, suitable for X-ray analysis, were obtained by adding [2.2.2]cryptand (376 mg, 0.91 mmol) to a suspension of **1** (481 mg, 0.91 mmol) in THF (5 mL) at –40 °C and further layering hexane. The crystals appeared colorless.

Remark: The [2.2.2]cryptand adduct is not stable at RT.

^{1}H NMR (400 MHz, DMF-d_7 δ 1.95 – 1.62 (m, 30H); **^{31}P{^{1}H} NMR** (162 MHz, DMF) δ 77.6; **^{13}C{^{1}H} NMR** (101 MHz, DMF) δ 41.3 (d, $^2J_{P\text{-}C}$ = 12.2 Hz), 38.4, 38.2 (d, $^1J_{P\text{-}C}$ = 22.2 Hz), 29.9 (d, $^3J_{P\text{-}C}$ = 7.8 Hz).

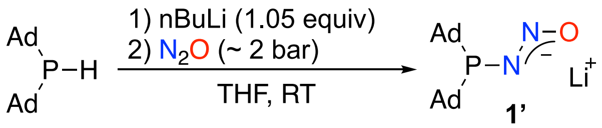

Compound 1’: A dried Schlenk flask equipped with a stir bar was charged with $HPAd_2$ (151 mg, 0.50 mmol) and THF (10 mL). The mixture was cooled to –40 °C and *n*-BuLi (1.6M in hexane, 0.33 mL, 0.53 mmol) was added dropwise. The mixture was allowed to warm to RT. After 15 min, the N_2 atmosphere was replaced by N_2O (3 vacuum/N_2O cycles) and the mixture was stirred under N_2O atmosphere (~ 2 bar). After 30 min, ^{31}P{^{1}H} NMR of an aliquot was measured and indicated the clean formation of **1’**.

{^{1}H}^{31}P NMR (162 MHz, THF) δ 77.5.

Remark: Crystallization attempts were unsuccessful.

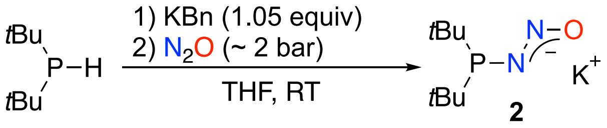

Compound 2: A dried Schlenk flask equipped with a stir bar was charged with HP*t*Bu$_2$ (29 mg, 0.25 mmol) and THF (1 mL). The mixture was cooled to –40 °C and benzyl potassium (34 mg, 0.26 mmol) was added. The mixture was allowed to warm to RT. After 15 min, the N_2 atmosphere was replaced by N_2O (3 vacuum/N_2O cycles) and the mixture was stirred under N_2O atmosphere (~ 2 bar). After 10 min, the solvent was removed under reduced pressure. The white crude was washed with hexane (1 mL) to give the product **2** as a white solid; yield: 86% (48 mg, 0.21 mmol).

Remark: Unreacted potassium di-*tert*-butylphosphanide (~10%) was present alongside compound **2** and could not be separated. Longer reaction time with N_2O resulted in the formation of unidentified side products.

Crystals, suitable for X-ray analysis, were obtained by adding [2.2.2]cryptand (0.25 mmol) to a suspension of **2** (0.25 mmol) in THF (1 mL) at –40 °C and further layering with hexane. The crystals appeared colorless.
Remark: The [2.2.2]cryptand adduct is not stable at RT.

^{1}H NMR (400 MHz, DMF) δ 1.04 (d, $^3J_{P\text{-}H}$ = 10.2 Hz, 18H); **^{31}P{^{1}H}** NMR (162 MHz, DMF) δ 78.8; **^{13}C{^{1}H}** NMR (101 MHz, DMF) δ 33.43 (d, $^1J_{P\text{-}C}$ = 22.2 Hz), 29.83 (d, $^2J_{P\text{-}C}$ = 14.4 Hz).

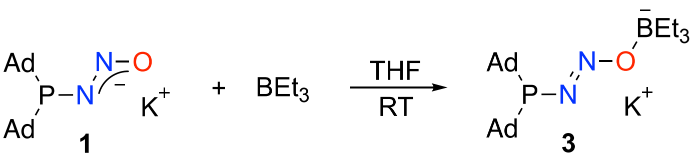

Compound 3: A dried scintillation vial equipped with a stir bar was charged with **1** (Ad_2PN_2OK • 0.5 THF, 21 mg, 50 μmol) and THF (1 mL). A solution of triethylborane (1M in THF, 50 μL, 50 μmol) was added dropwise to the mixture at RT. After 1 min, the mixture appeared homogeneous and colorless. After 15 min, the solvent was removed under reduced pressure. The white crude was washed with cold hexane (2 x 0.5 mL) to give the pure product **3** as a white solid; yield: 96% (23 mg, 48 μmol).
Crystals, suitable for X-ray analysis, were obtained by layering hexane onto a concentrated solution of **3** in THF at –40 °C. The crystals appeared colorless.

^{1}H NMR (400 MHz, THF) δ 2.05 – 1.64 (m, 30H), 0.71 (t, $^3J_{H\text{-}H}$ = 7.7 Hz, 9H), 0.28 (q, $^3J_{H\text{-}H}$ = 7.7 Hz, 6H); **^{31}P{^{1}H} NMR** (162 MHz, THF) δ 84.2; **^{11}B{^{1}H}** NMR (128 MHz, THF) δ 5.08; **^{13}C{^{1}H} NMR** (101 MHz, THF) δ 40.9 (d, $^2J_{P\text{-}C}$ = 12.2 Hz), 39.0 (d, $^1J_{P\text{-}C}$ = 26.4 Hz), 38.3, 30.0 (d, $^3J_{P\text{-}C}$ = 7.8 Hz), 16.3, 11.3.

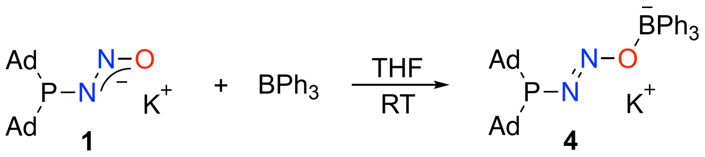

Compound 4: A dried scintillation vial equipped with a stir bar was charged with **1** (Ad_2PN_2OK • 0.5 THF, 21 mg, 50 μmol) and THF (1 mL). Triphenylborane (12 mg, 50 μmol) was added to the mixture at RT. After 1 min, the mixture appeared homogeneous and colourless. After 15 min, the solvent was removed under reduced pressure to give product **4** as a white solid; yield: 96% (30 mg, 48 μmol).

^{1}H NMR (400 MHz, THF) δ 7.48 – 7.17 (m, 6H), 7.14 – 6.80 (m, 9H), 2.14 – 1.51 (30H); **^{31}P{^{1}H} NMR** (162 MHz, THF) δ 87.6; **^{11}B{^{1}H}** NMR (128 MHz, THF) δ 0.32; **^{13}C{^{1}H} NMR** (101 MHz, THF) δ 159.6, 135.3, 126.7, 124.1, 40.7 (d, $^2J_{P-C}$ = 11.7 Hz), 39.1 (d, $^1J_{P-C}$ = 25.3 Hz), 38.1, 29.9 (d, $^3J_{P-C}$ = 8.0 Hz).

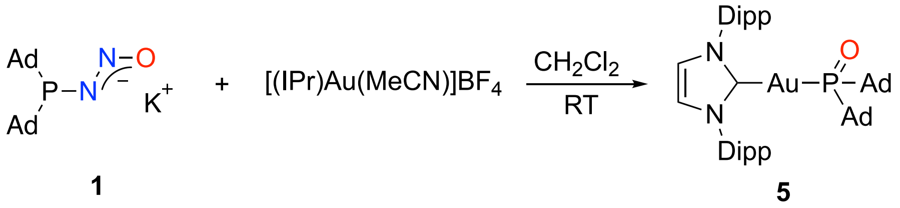

Complex 5: A dried scintillation vial equipped with a stir bar was charged with **1** (Ad_2PN_2OK • 0.5 THF, 35 mg, 90 μmol) and THF (1 mL). [(IPr)Au(MeCN)]BF_4 (43 mg, 60 μmol) was added at RT. After 16 hours, the solution was filtered, and the solvent was removed under reduced pressure. The crude was redissolved with DCM (2 mL), the solution was filtered, and the solvent was removed under reduced pressure to afford complex **5** as a white solid; yield: 76% (42 mg 46 μmol). Crystals, suitable for X-ray analysis, were obtained by layering hexane onto a concentrated solution of **5** in DCM at –40 °C.

^{1}H NMR (500 MHz, CD_2Cl_2) δ 7.50 – 7.45 (2H), 7.33 – 7.30 (4H), 7.25 (s, 2H), 2.60 (p, J = 6.9 Hz, 4H), 1.76 – 1.47 (m, 30H), 1.36 (d, J = 6.9 Hz, 12H), 1.22 (d, J = 6.9 Hz, 12H); **^{31}P{^{1}H} NMR** (162 MHz, CD_2Cl_2) δ 114.6; **^{13}C{^{1}H}** NMR (126 MHz, CD_2Cl_2) δ 202.1 (d, $^2J_{P-C}$ = 122.3 Hz), 146.2, 134.6, 130.7, 124.3, 123.7 (d, $^4J_{P-C}$ =

3.3 Hz), 42.00 (d, $^{1}J_{P\text{-}C}$ = 30.7 Hz), 38.97 (d, $^{4}J_{P\text{-}C}$ = 3.1 Hz), 37.65, 29.20, 29.05 (d, $^{3}J_{P\text{-}C}$ = 9.1 Hz), 24.49 (d, $^{2}J_{P\text{-}C}$ = 25.5 Hz).

HRMS (ESI/QTOF) *m/z*: $[M]^+$ Calcd for $C_{47}H_{67}AuN_2OP^+$ 903.4651; Found 903.4679.

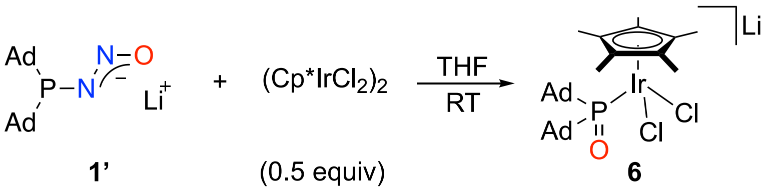

1’ (0.5 equiv) **6**

Complex 6: A dried scintillation vial equipped with a stir bar was charged with a solution of **1’** in THF (0.05 M, 2 mL). $[Cp^*IrCl_2]_2$ (40 mg, 50 μmol) was added at RT. After 4 h, the solution was filtered and cooled to –40 °C overnight to afford complex **6** as a red crystalline solid; yield: 71% (26 mg, 36 μmol). Crystals, suitable for X-ray analysis, were obtained by slow evaporation of a solution of **6** in THF at RT.

^{1}H NMR (400 MHz, CD_2Cl_2) δ 2.13 – 2.04 (m, 12H), 1.98 – 1.92 (m, 6H), 1.78 – 1.65 (m, 12H), 1.62 (d, $^{4}J_{P\text{-}H}$ = 1.6 Hz, 15H); **$P^{31}\{^1H\}$ NMR** (162 MHz, CD_2Cl_2) δ 89.8; **$^{13}C\{^1H\}$ NMR** (101 MHz, CD_2Cl_2) δ 91.0 (d, $^{3}J_{P\text{-}C}$ = 2.8 Hz), 47.1 (d, $^{1}J_{P\text{-}C}$ = 26.6 Hz), 39.3, 37.6, 29.4 (d, $^{2}J_{P\text{-}C}$ = 8.7 Hz), 11.0.

HRMS (ESI/QTOF) *m/z*: $[M - 2Cl]^+$ Calcd for $C_{30}H_{47}IrOP^+$ 647.2988; Found 647.2981.

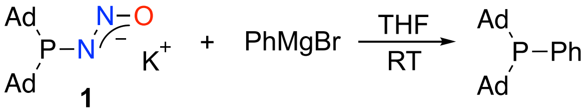

1

Diadamantylphenylphosphine: A dried Schlenk flask equipped with a stir bar was charged with **1** (Ad_2PN_2OK • 0.5 THF, 84 mg, 0.20 mmol) and THF (2 mL). Phenyl magnesium bromide (3M in Et_2O, 0.2 mL, 0.60 mmol) was added dropwise at RT. After 12 h, the mixture was cooled to 0 °C and quenched with sat. aq NH_4Cl (1 mL). Et_2O (5 mL) was added. The phases were separated, and the aqueous phase was extracted with Et_2O (2 × 3 mL). The combined organic phases were dried ($MgSO_4$), and solvents were evaporated under reduced pressure and EtOAc (5 mL) was added to the crude

product. The mixture was then filtered through a pad of silica gel and washed with EtOAc (5 mL). The solvent was removed under reduced pressure to give the pure product as a white solid; yield: 72% (54 mg, 0.14 mmol).

^{1}H NMR (400 MHz, CD_2Cl_2) δ 7.65 – 7.05 (m, 5H), 1.94 – 1.53 (m, 30H); **^{31}P{^{1}H} NMR** (162 MHz, CD_2Cl_2) δ 41.4. The spectroscopical data are in accordance with the reported data.[4]

3. Control experiments

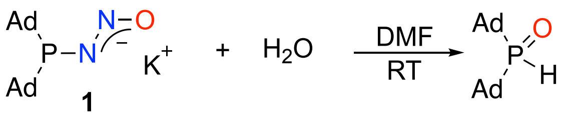

Inside a glovebox, a J-Young NMR tube was charged with **1** (Ad_2PN_2OK • 0.5 THF, 21 mg, 50 μmol) in DMF (1 mL). Outside the glovebox, degassed water was added (50 μL) at RT and intense bubbling was immediately observed (release of N_2). The *in situ* ^{31}P NMR measurement indicated the complete conversion of **1** into the phosphine oxide (δ 58.6 (d, $^1J_{P\text{-}H}$ = 426.1 Hz)). The chemical shift is in agreement with the reported value.[5]

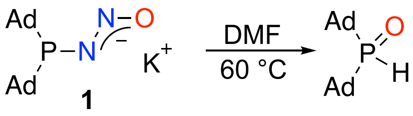

Inside a glovebox, a J-Young NMR tube was charged with **1** (Ad_2PN_2OK • 0.5 THF, 21 mg, 50 μmol) and DMF (1 mL). The mixture was heated to 60 °C and followed by *in situ* ^{31}P NMR. After 2 h, **1** was fully converted to the phosphine oxide (δ 58.6 (d, $^1J_{P\text{-}H}$ = 426.1 Hz)). The chemical shift is in agreement with the reported value.[5]

4. NMR spectra

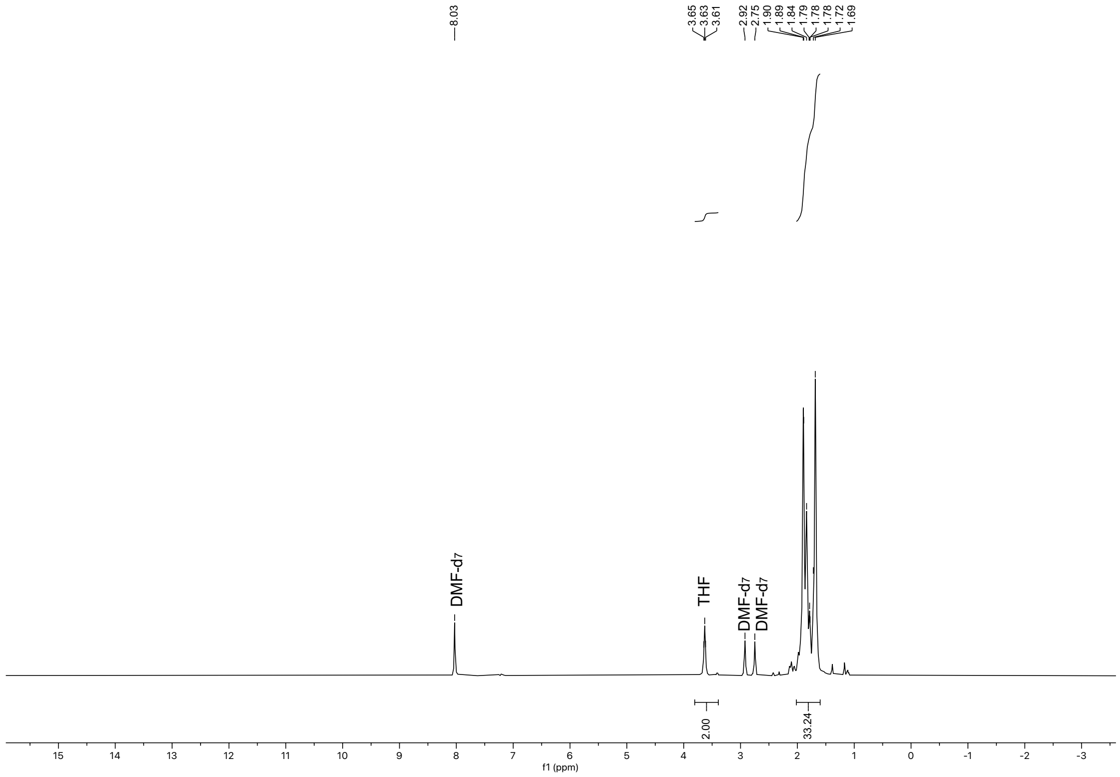

Figure S1. ^{1}H NMR (400 MHz, DMF-d_7) spectrum of compound **1**.

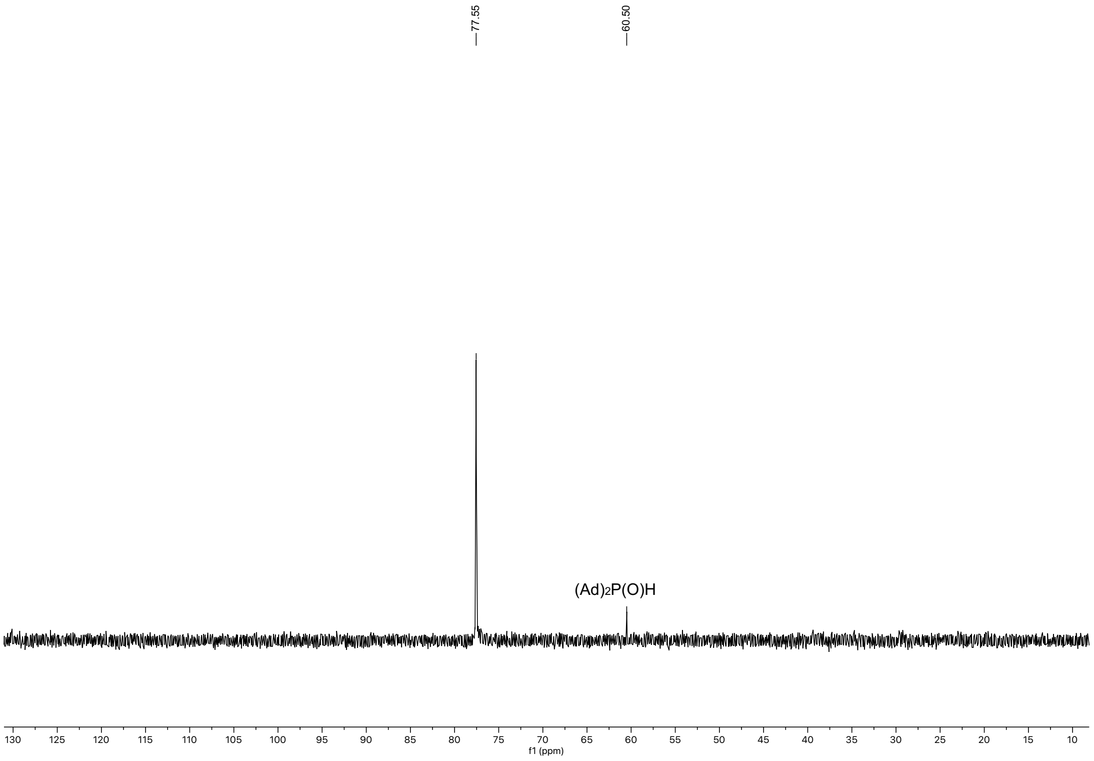

Figure S2. $^{31}P\{^{1}H\}$ NMR (162 MHz, DMF-d_7) spectrum of compound **1**.

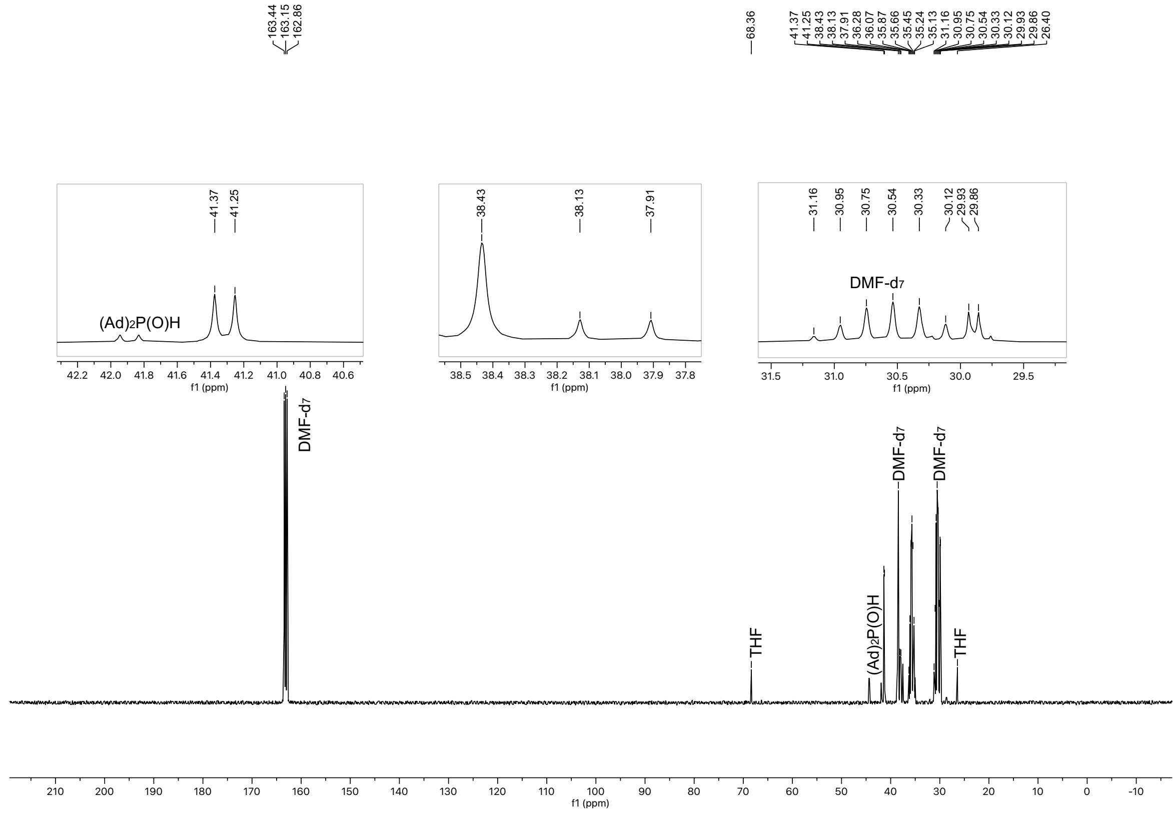

Figure S3. $^{13}C\{^{1}H\}$ NMR (101 MHz, DMF-d_7) spectrum of compound **1**.

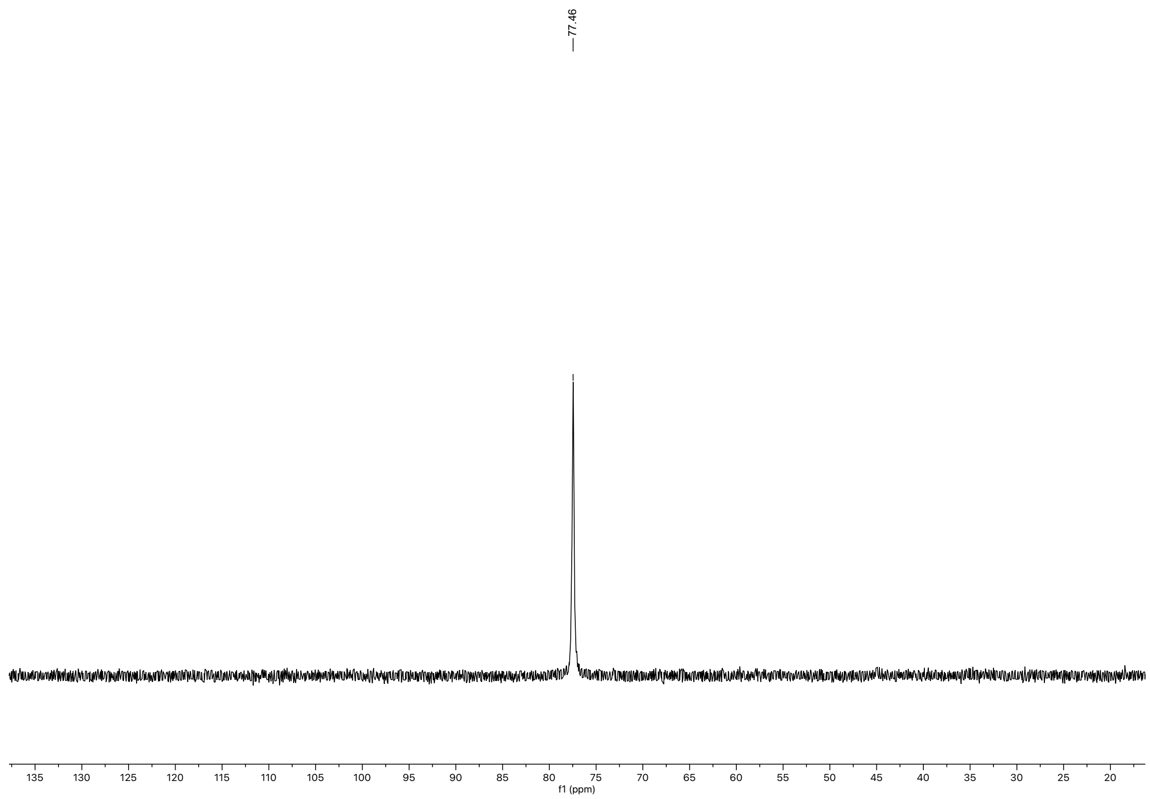

Figure S4. $^{31}P\{^{1}H\}$ NMR (162 MHz, DMF-d_7) spectrum of compound **1'**.

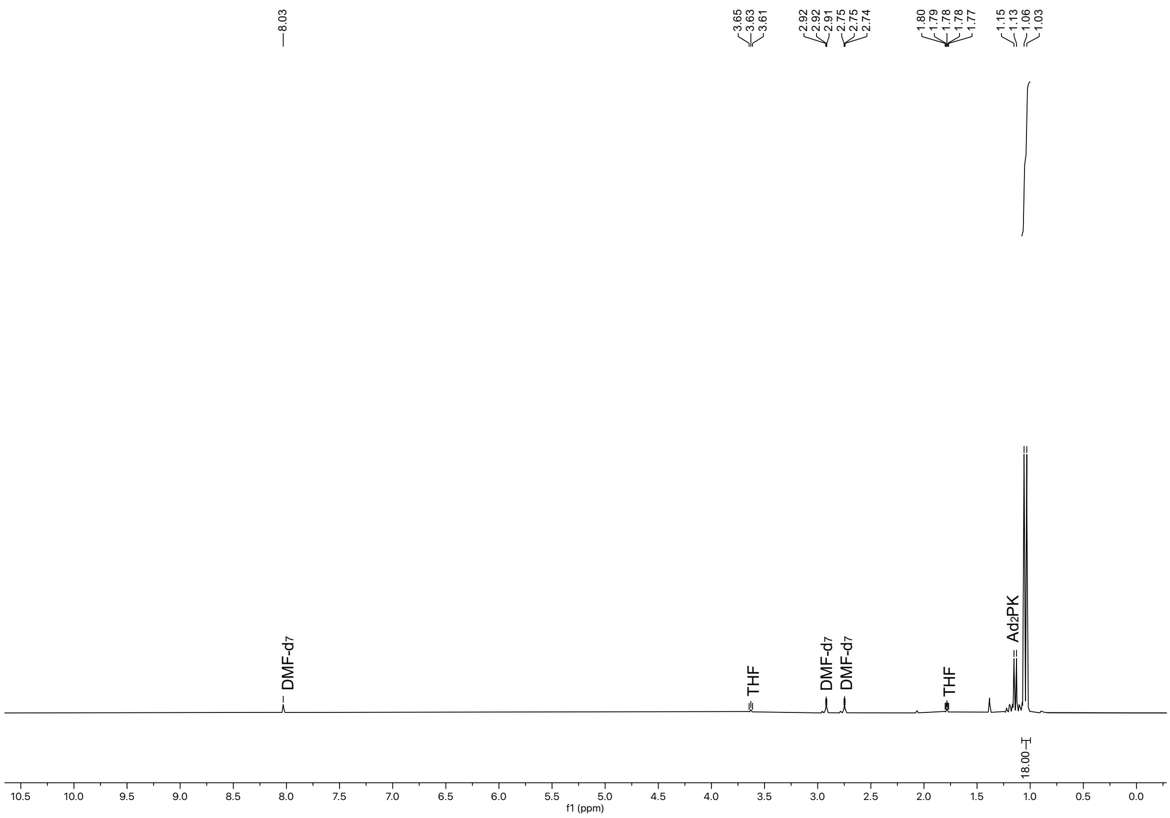

Figure S5. ^{1}H NMR (400 MHz, DMF-d_7) spectrum of compound **2**.

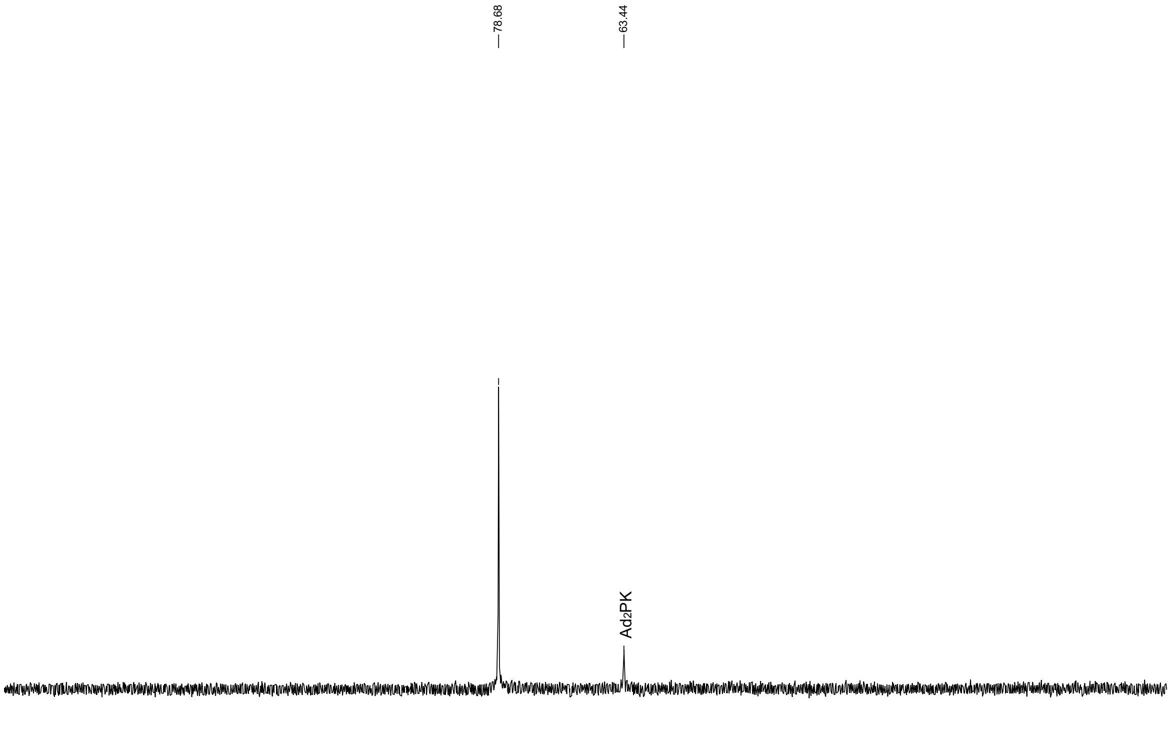

Figure S6. $^{31}P\{^{1}H\}$ NMR (162 MHz, DMF-d_7) spectrum of compound **2**.

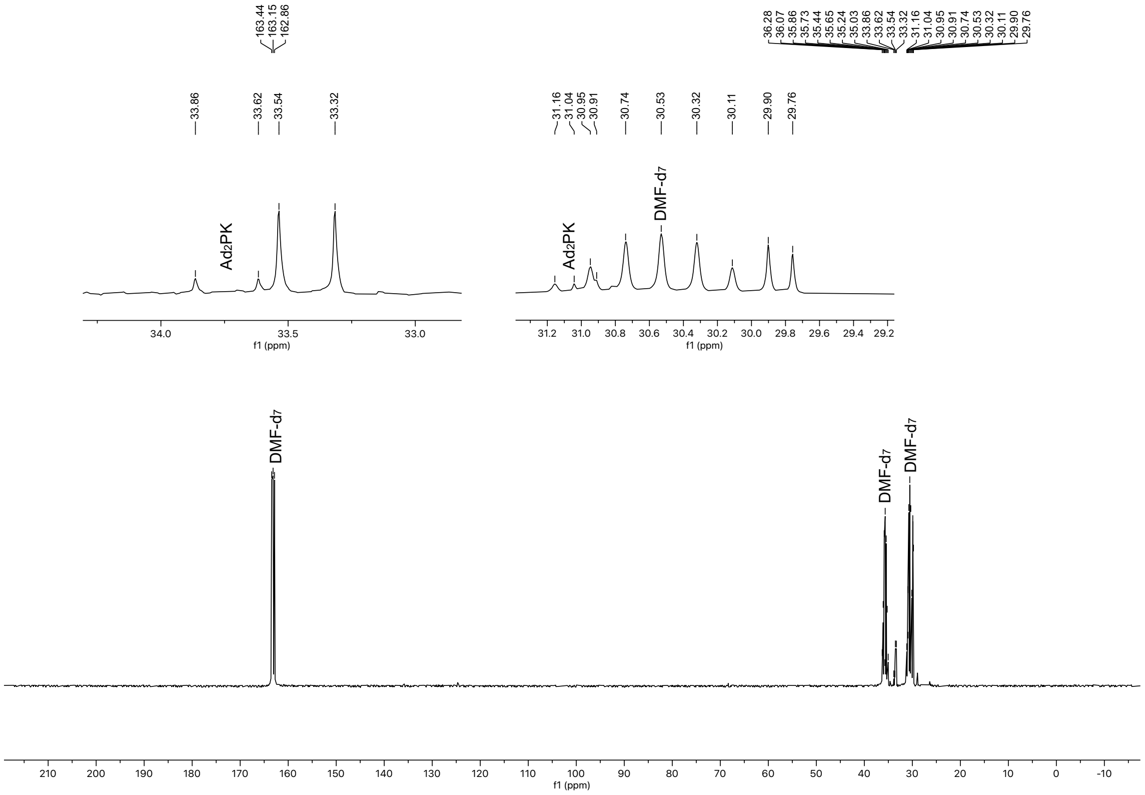

Figure S7. $^{13}C\{^{1}H\}$ NMR (101 MHz, DMF-d_7) spectrum of compound **2**.

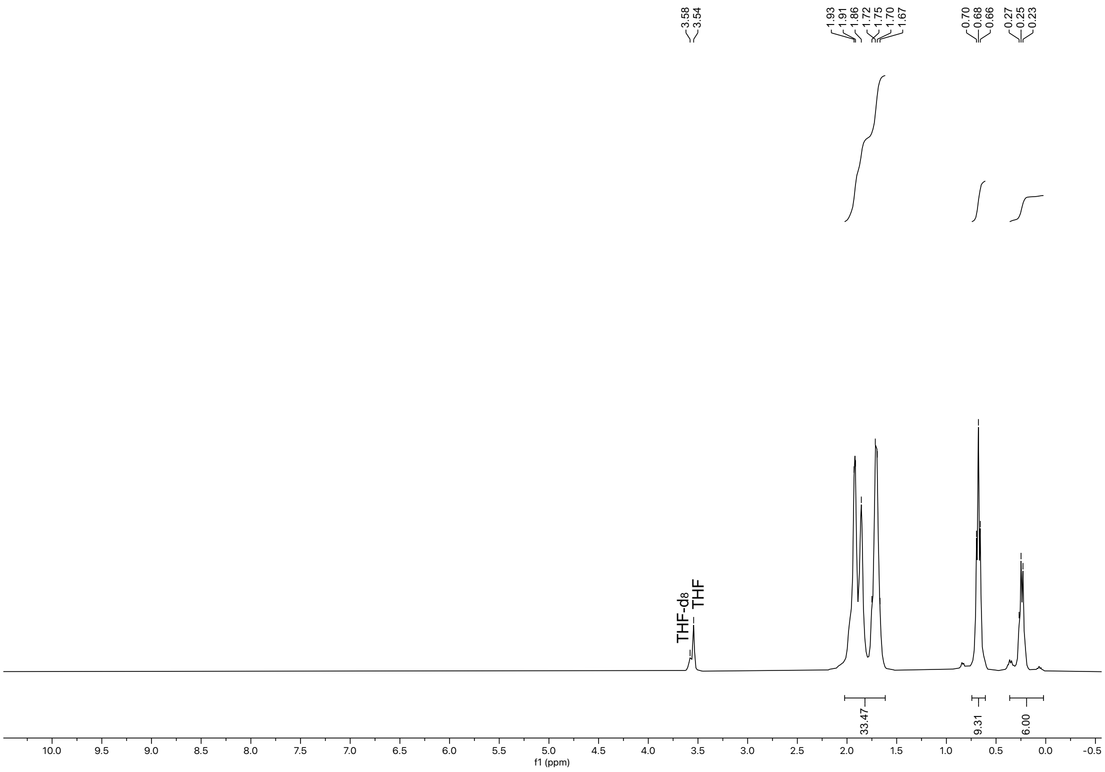

Figure S8. ^{1}H NMR (400 MHz, THF-d_8) spectrum of compound **3**.

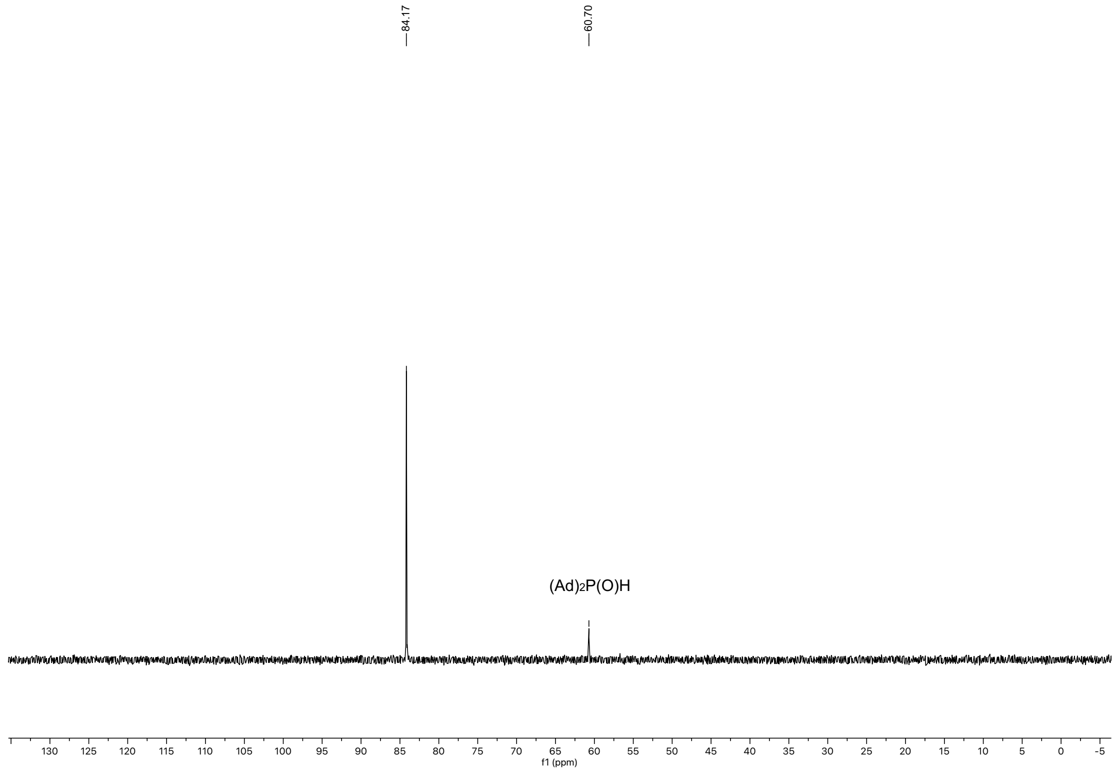

Figure S9. $^{31}P\{^{1}H\}$ NMR (162 MHz, THF-d_8) spectrum of compound **3**.

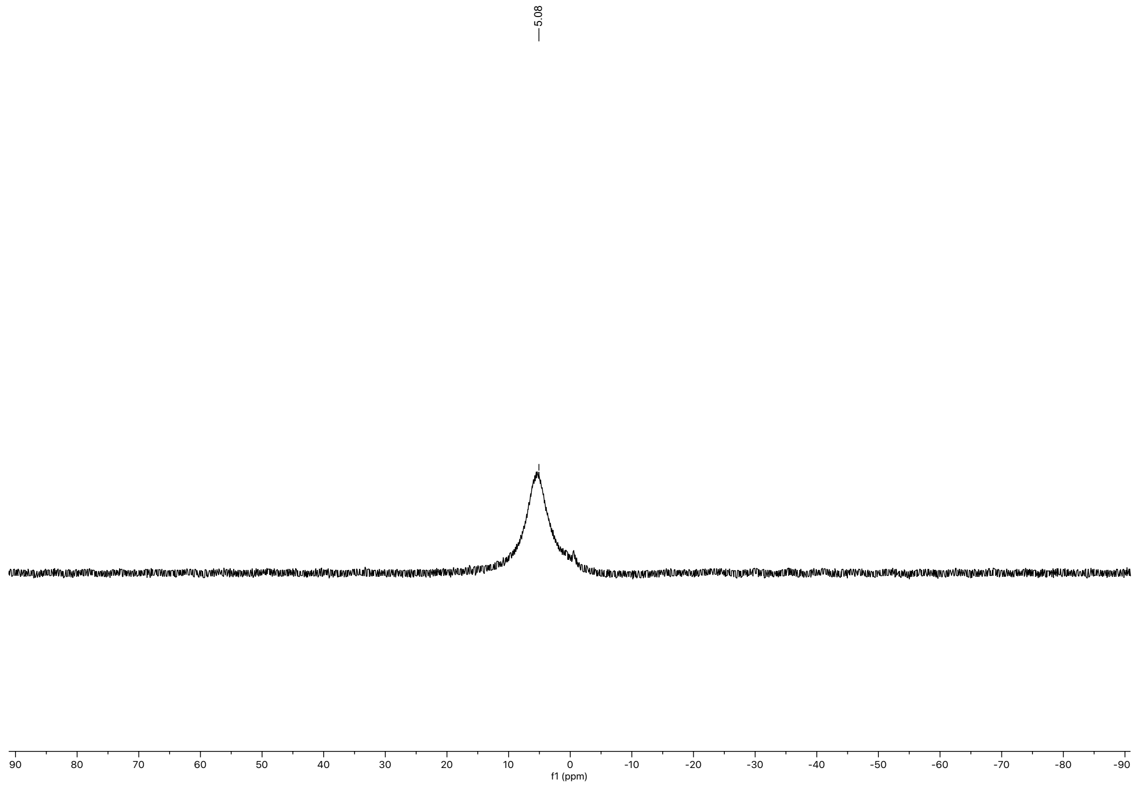

Figure S10. $^{11}B\{^{1}H\}$ NMR (128 MHz, THF-d_8) spectrum of compound **3**.

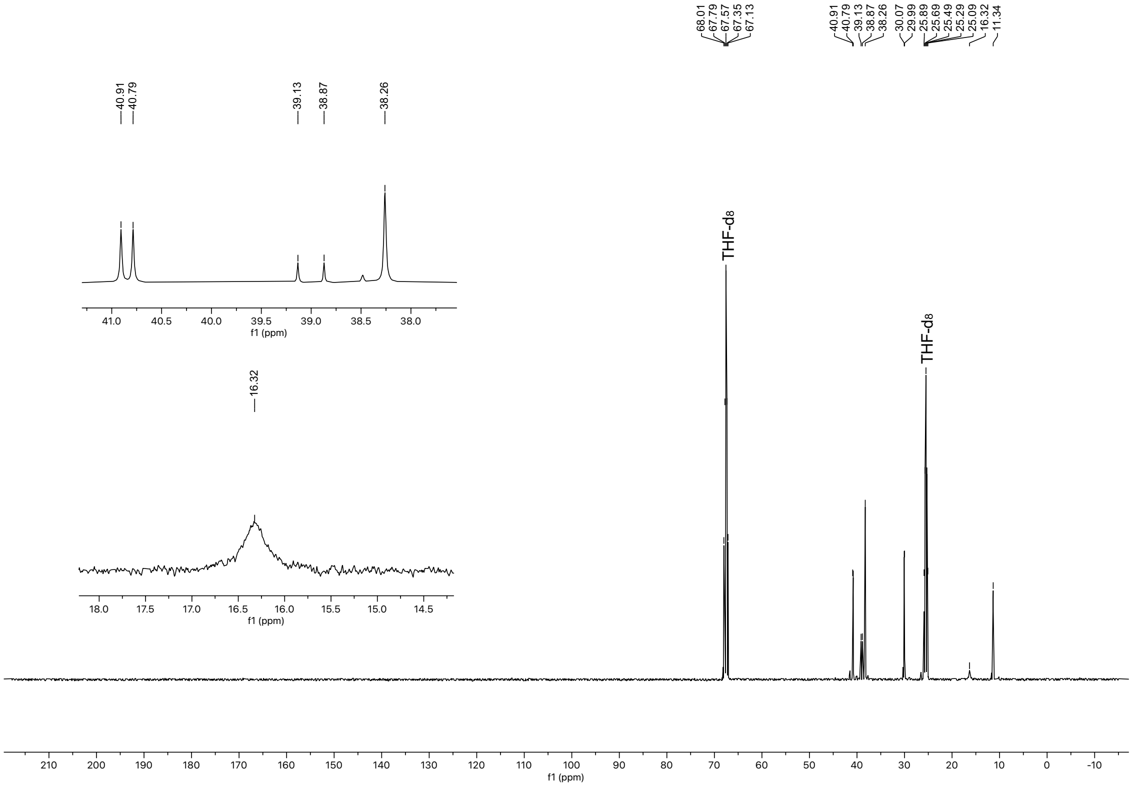

Figure S11. $^{13}C\{^{1}H\}$ NMR (101 MHz, THF-d_8) spectrum of compound **3**.

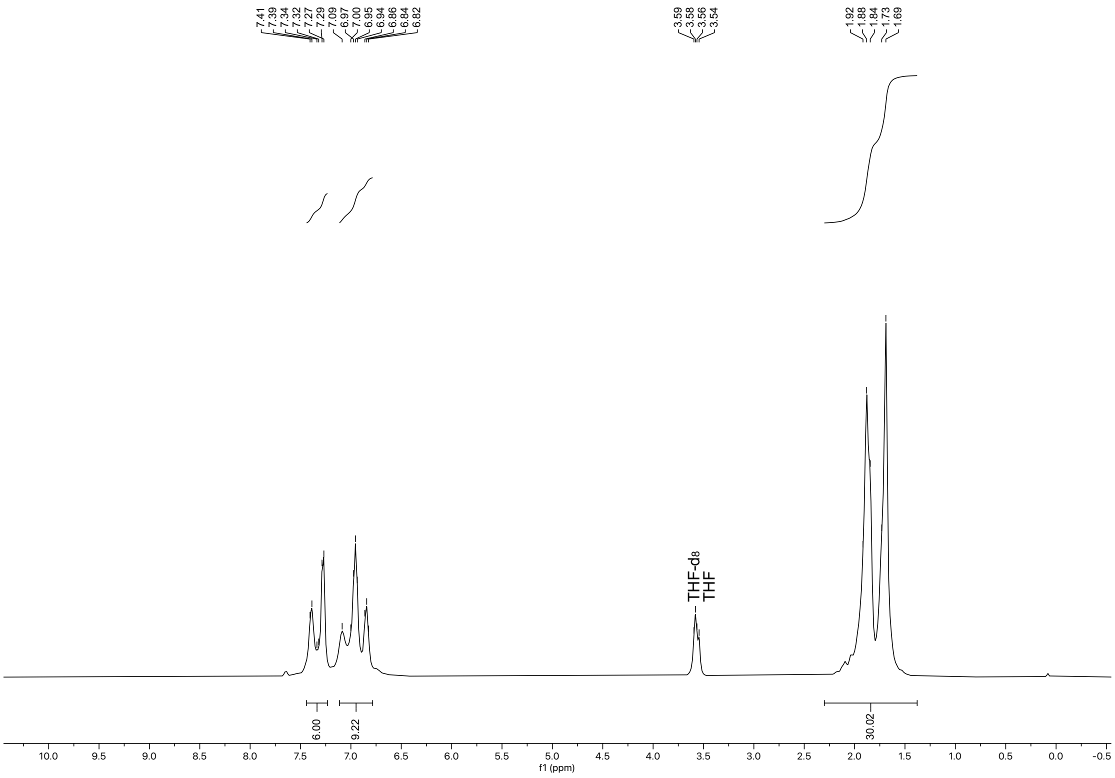

Figure S12. ^{1}H NMR (400 MHz, THF-d_8) spectrum of compound **4**.

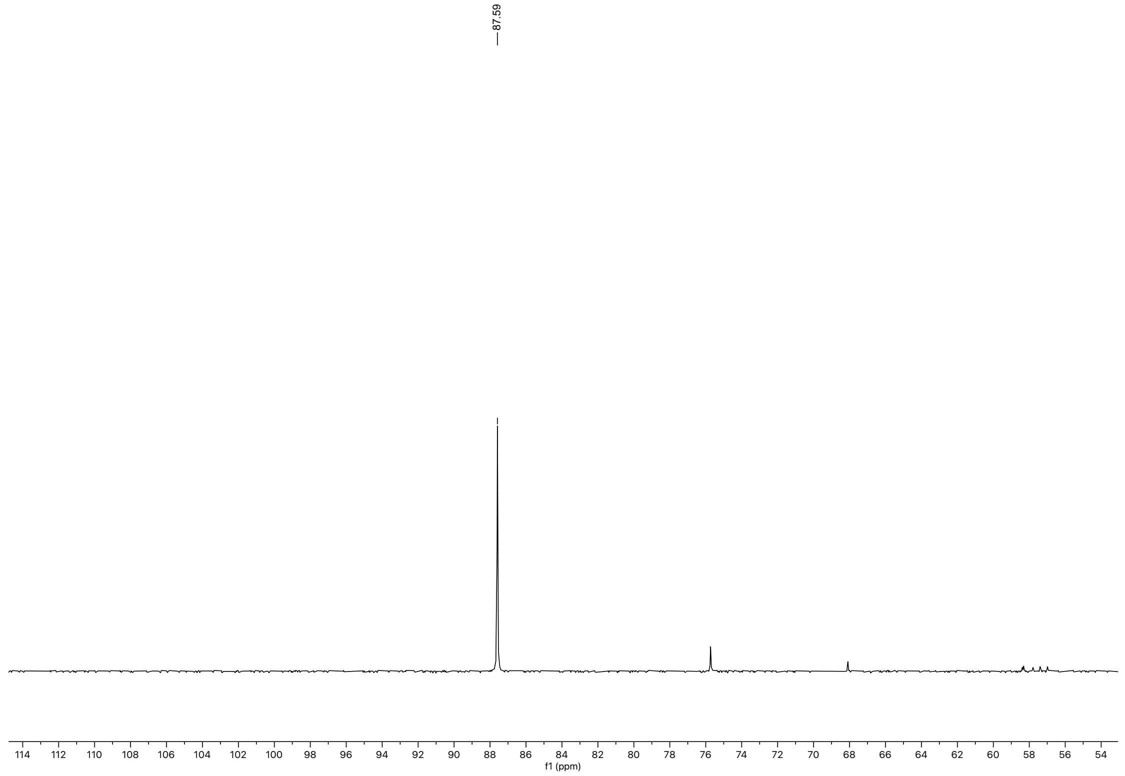

Figure S13. $^{31}P\{^{1}H\}$ NMR (162 MHz, THF-d_8) spectrum of compound **4**.

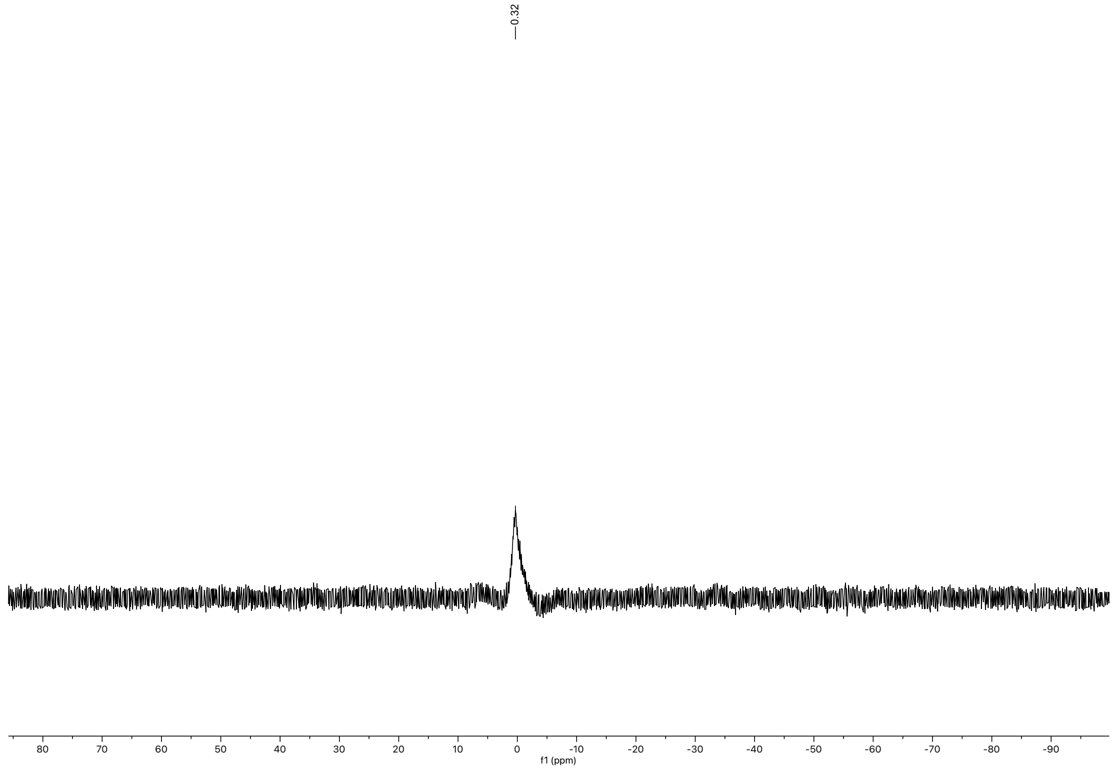

Figure S14. $^{11}B\{^{1}H\}$ NMR (128 MHz, THF-d_8) spectrum of compound **4**.

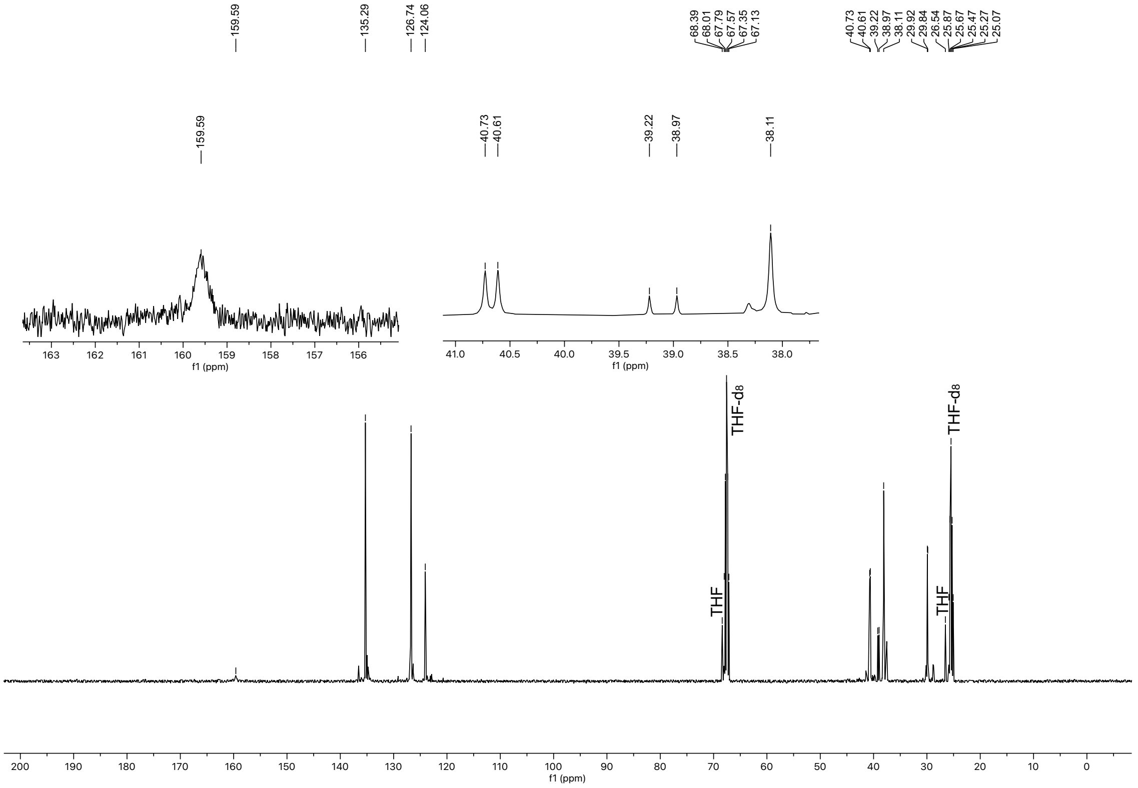

Figure S15. $^{13}C\{^1H\}$ NMR (101 MHz, THF-d$_8$) spectrum of compound **4**.

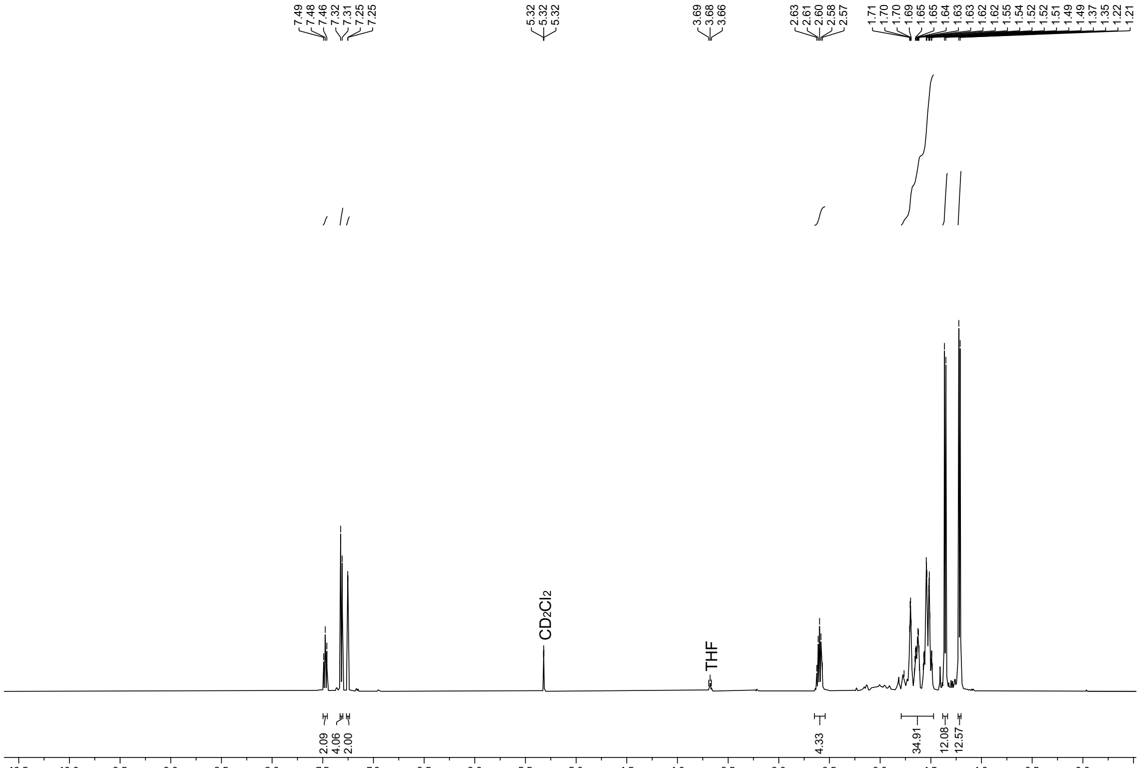

Figure S16. ^1H NMR (400 MHz, CD_2Cl_2) spectrum of compound **5**.

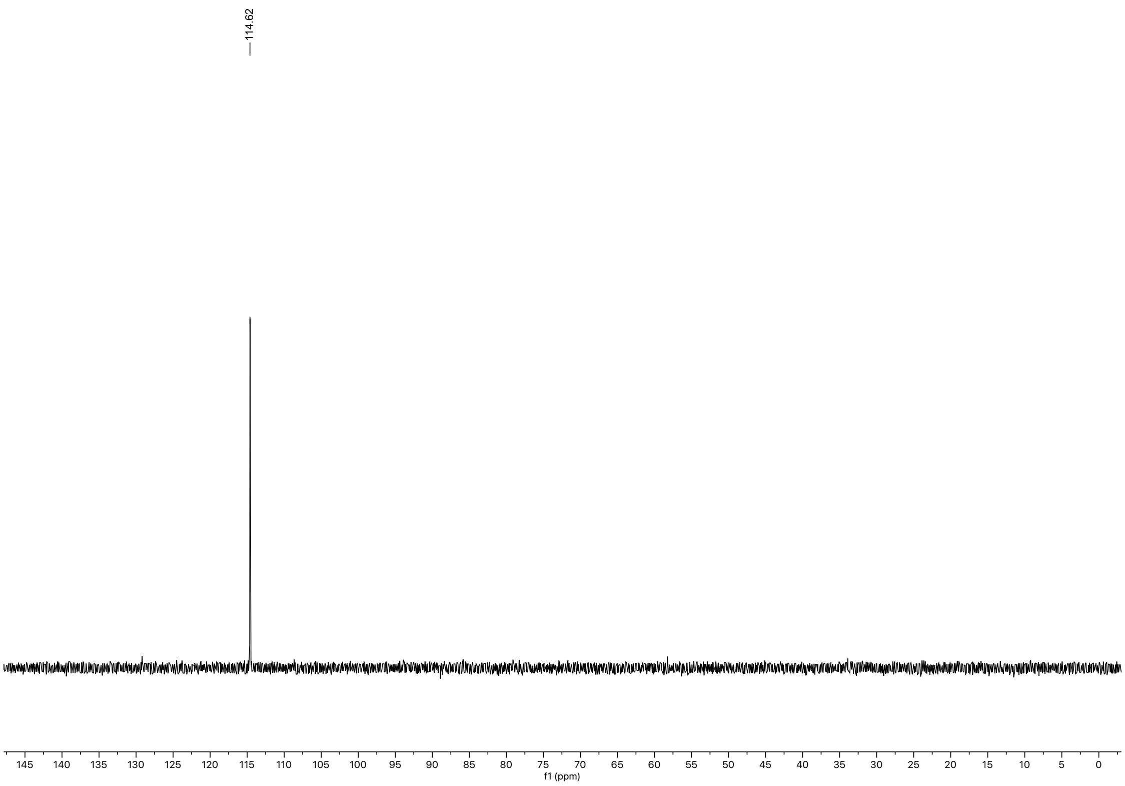

Figure S17. $^{31}P\{^1H\}$ NMR (162 MHz, CD_2Cl_2) spectrum of compound **5**.

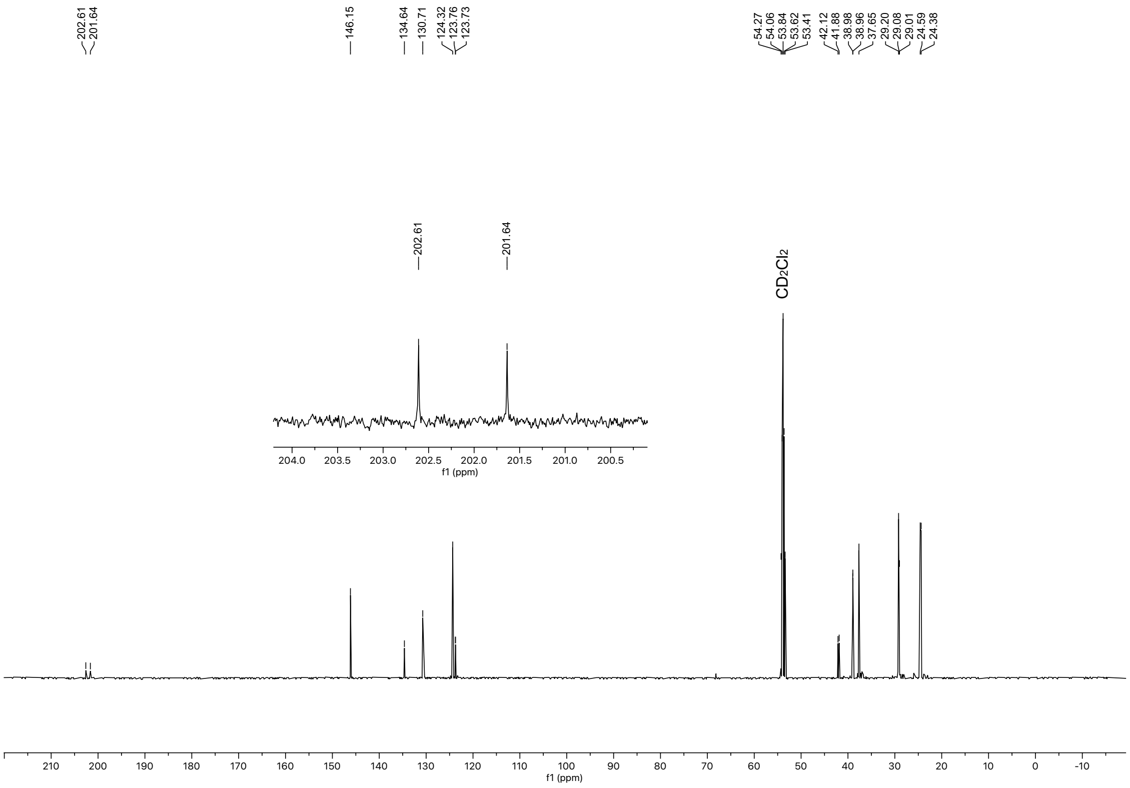

Figure S18. $^{13}C\{^1H\}$ NMR (126 MHz, CD_2Cl_2) spectrum of compound **5**.

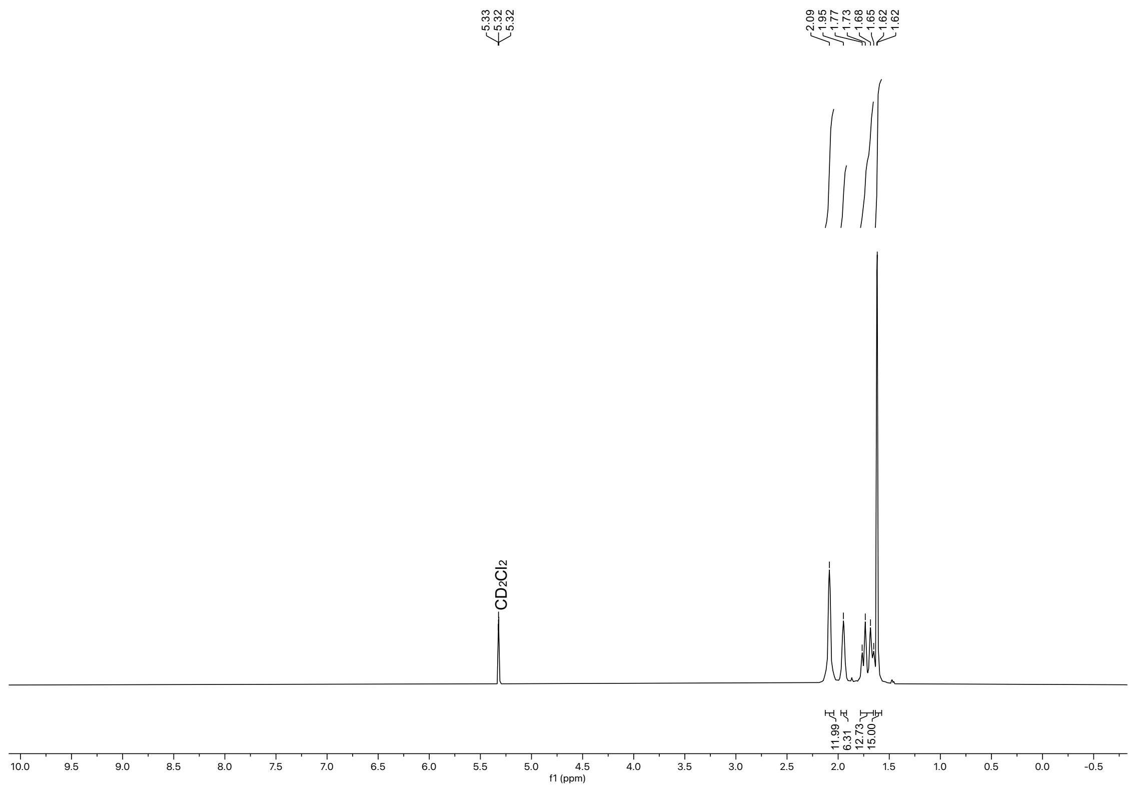

Figure S19. ^1H NMR (400 MHz, CD_2Cl_2) spectrum of compound **6**.

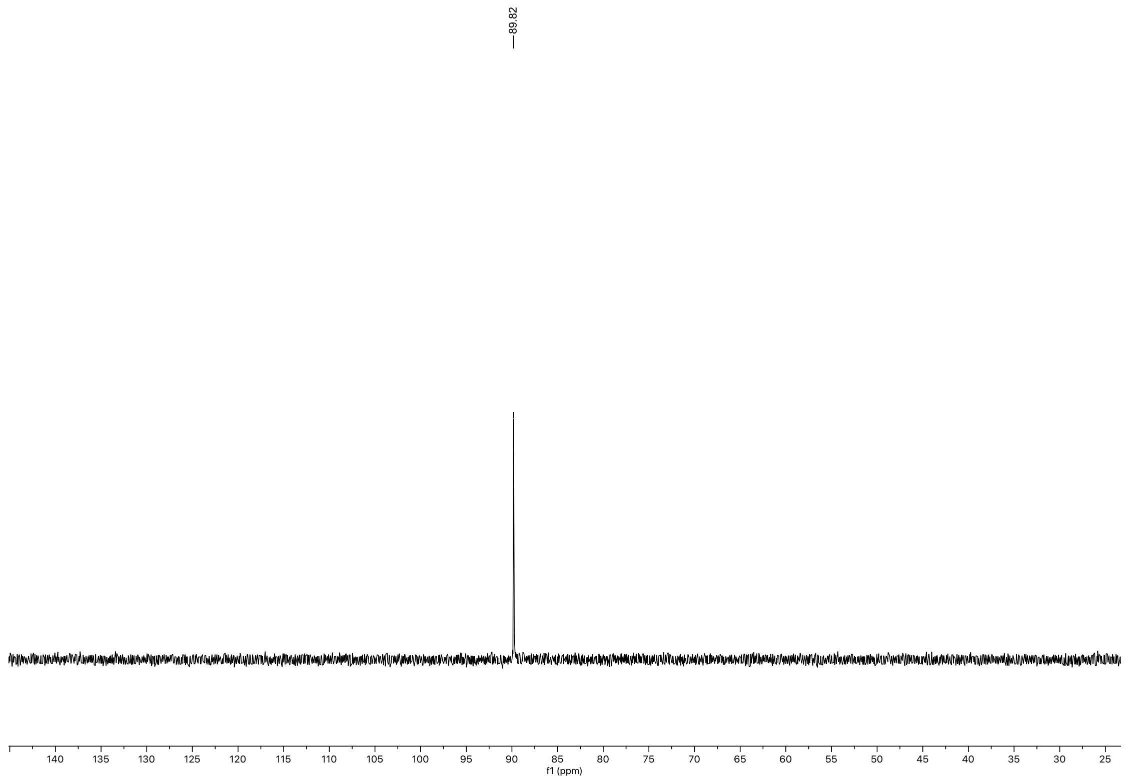

Figure S20. $^{31}P\{^1H\}$ NMR (162 MHz, CD_2Cl_2) spectrum of compound **6**.

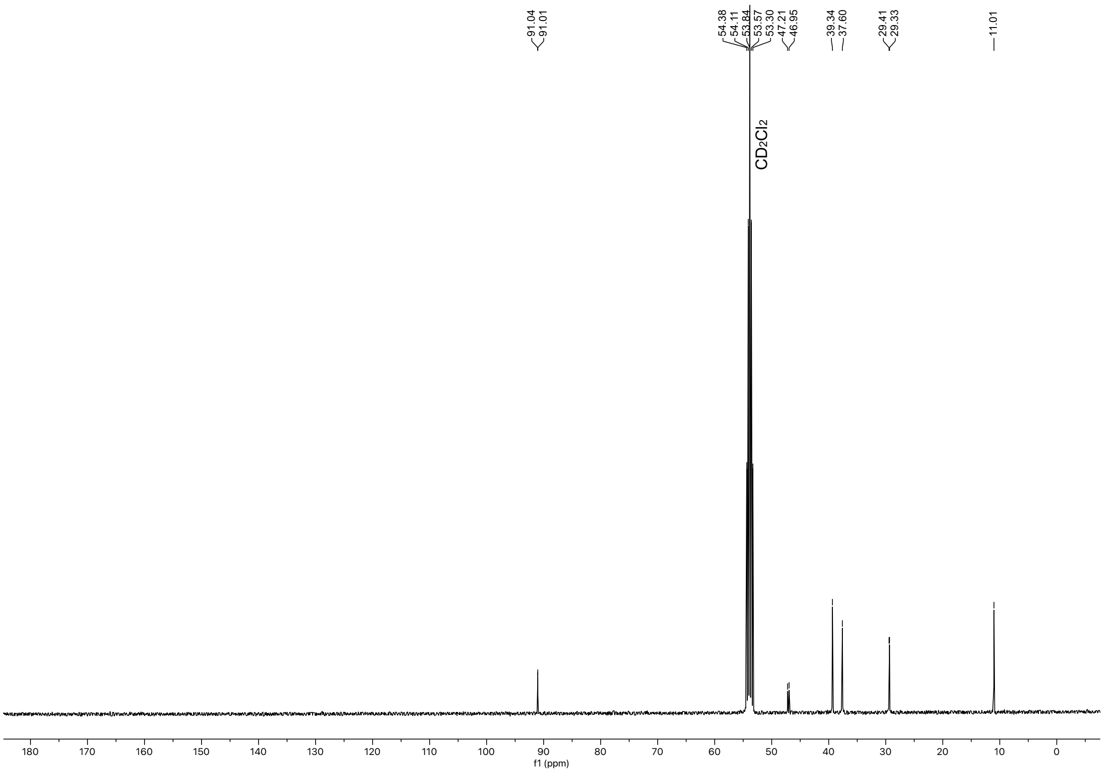

Figure S21. $^{13}C\{^{1}H\}$ NMR (101 MHz, CD_2Cl_2) spectrum of compound **6**.

5. Single crystal X-ray analyses

Compound **1**

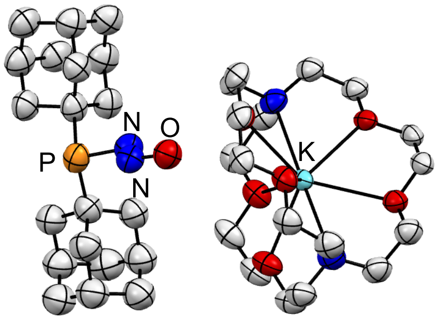

Figure S22. Molecular structure of compound **1** in the crystal. Thermal ellipsoids are at 40% probability and hydrogen atoms are not depicted. Selected bond lengths (Å) and angles (°): P–N 1.723(7), N–N 1.257(9), N–O 1.260(8), P–N–N 110.6(6), N–N–O 115.4(7).

Structure Quality Indicators

**Reflections:**	d min (CuKα) 2Θ=100.9° 1.00	I/σ(I) 11.7	Rint m=6.40 11.84%	Full 100.9° 99.6
**Refinement:**	Shift 0.000	Max Peak 0.5	Min Peak -0.4	GooF 1.081

A colorless plate-shaped crystal with dimensions 0.17 × 0.05 × 0.02 mm^3 was mounted. Data were collected using a XtaLAB Synergy R, DW system, HyPix-Arc 150 diffractometer operating at T = 140.00(10) K. Data were measured using ω scans with CuK_α radiation. The diffraction pattern was indexed, and the total number of runs and images was based on the strategy calculation from the program CrysAlisPro[6] system (CCD 43.134a 64-bit (release 09-08-2024)). The maximum resolution achieved was Θ = 50.429° (1.00 Å). The unit cell was refined using CrysAlisPro 1.171.43.135a (Rigaku OD, 2024) on 8153 reflections, 26% of the observed reflections. Data reduction, scaling and absorption corrections were performed using CrysAlisPro 1.171.43.135a (Rigaku OD, 2024). The final completeness is 99.60 % out to 50.429° in Θ. A Gaussian absorption correction was performed using CrysAlisPro 1.171.43.135a (Rigaku Oxford Diffraction, 2024) Numerical absorption correction based on Gaussian integration over a multifaceted crystal model. Empirical absorption correction using spherical harmonics as implemented in SCALE3 ABSPACK scaling algorithm. The absorption coefficient μ of this material is 1.715 mm^{-1} at this wavelength (λ = 1.54184Å) and the minimum and maximum transmissions are 0.697 and 1.000. The structure was solved in the space group *P*2/*n* (# 13) by the ShelXT 2018/2 (Sheldrick, 2015) structure solution program using dual methods and refined by full matrix least squares minimisation on $\boldsymbol{F}^2$ using version 2019/3 of ShelXL 2019/3 (Sheldrick, 2015).[7] All non-hydrogen atoms were refined anisotropically. Hydrogen atom positions were calculated geometrically and refined using the riding model. A solvent mask was calculated, and 294 electrons were found in a volume of 1218$Å^3$ in one void per unit cell. This is consistent with the presence of 2 THF per Asymmetric Unit which account for 320 electrons per unit cell. There is a single formula unit in the asymmetric unit, which is represented by the reported sum formula. In other words: Z is 4 and Z' is 1.

The moiety formula is $C_{18}H_{36}KN_2O_6$, $C_{20}H_{30}N_2OP$, $2[C_4H_8O]$.

Table S1. Crystal data and structure refinement for **1**.

Compound	ga-194-1
Formula	$C_{46}H_{82}KN_4O_9P$
$D_{calc.}$/ g cm^{-3}	1.234
μ/mm^{-1}	1.715
Formula Weight	905.22
Colour	colorless
Shape	plate-shaped
Size/mm^3	0.17×0.05×0.02
T/K	140.00(10)
Crystal System	monoclinic
Space Group	*P*2/*n*
a/Å	11.2324(5)
b/Å	11.8181(6)
c/Å	36.8937(13)
$\alpha/^\circ$	90
$\beta/^\circ$	95.713(4)
$\gamma/^\circ$	90
V/Å^3	4873.1(4)
Z	4
Z'	1
Wavelength/Å	1.54184
Radiation type	Cu$K\alpha$
$\Theta_{min}/^\circ$	2.407
$\Theta_{max}/^\circ$	50.429
Measured Refl's.	31097
Indep't Refl's	5095
Refl's I≥2σ(I)	3243
R_{int}	0.1184
Parameters	460
Restraints	861
Largest Peak/e Å^{-3}	0.467
Deepest Hole/e Å^{-3}	–0.407
GooF	1.081
wR_2 (all data)	0.3123
wR_2	0.2896
R_1 (all data)	0.1360
R_1	0.0997
CCDC number	2473516

Compound **2**

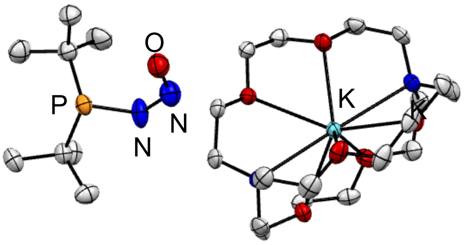

Figure S23. Molecular structure of compound **2** in the crystal. Thermal ellipsoids are at 40% probability and hydrogen atoms are not depicted. Selected bond lengths (Å) and angles (°): P–N 1.775(3), N–N 1.274(5), N–O 1.276(5), P–N–N 118.3(3), N–N–O 118.4(4).

Structure Quality Indicators

**Reflections:**	d min (CuKα) 2Θ=145.5° 0.81	I/σ(I) 12.6	Rint m=2.67 7.42%	Full 135.4° 98% to 145.5° 99.9
**Refinement:**	Shift 0.000	Max Peak 0.4	Min Peak -0.6	GooF 1.016

A colorless plate-shaped crystal with dimensions 0.13 × 0.10 × 0.02 mm^3 was mounted. Data were collected using a SuperNova, Dual, Cu at home/near, AtlasS2 diffractometer operating at T = 140.00(10) K. Data were measured using ω scans with CuK_α radiation. The diffraction pattern was indexed and the total number of runs and images was based on the strategy calculation from the program CrysAlisPro[6] system (CCD 44.111a 64-bit (release 13-05-2025)). The maximum resolution that was achieved was Θ = 72.735° (0.81 Å). The unit cell was refined using CrysAlisPro 1.171.44.111a (Rigaku OD, 2025) on 3620 reflections, 23% of the observed reflections. Data reduction, scaling and absorption corrections were performed using CrysAlisPro 1.171.44.111a (Rigaku OD, 2025). The final completeness is 99.90 % out to 72.735° in Θ. A Gaussian absorption correction was performed using CrysAlisPro 1.171.44.111a (Rigaku Oxford Diffraction, 2025) Numerical absorption correction based on Gaussian integration over a multifaceted crystal model. Empirical absorption correction using spherical harmonics as implemented in SCALE3 ABSPACK scaling algorithm. The absorption coefficient μ of this material is 2.298 mm^{-1} at this wavelength (λ = 1.54184Å) and the minimum and maximum transmissions are 0.760 and 1.000. The structure was solved in the space group $P2_1/c$ (# 14) by the ShelXT 2018/2 (Sheldrick, 2015) structure solution program using dual methods and refined by full matrix least squares minimisation on F^2 using version 2019/3 of ShelXL 2019/3 (Sheldrick, 2015).[7] All non-hydrogen atoms were refined anisotropically. Hydrogen atom positions were calculated geometrically and refined using the riding model. There is a single formula unit in the asymmetric unit, which is represented by the reported sum formula. In other words: Z is 4 and Z' is 1. The moiety formula is $C_{18}H_{36}KN_2O_6$, $C_8H_{18}N_2OP$.

Table S2. Crystal data and structure refinement for **2**.

Compound	ga-306
Formula	$C_{26}H_{54}KN_4O_7P$
$D_{calc.}$/ g cm^{-3}	1.249
μ/mm^{-1}	2.298
Formula Weight	604.80
Colour	colorless
Shape	plate-shaped
Size/mm^3	0.13×0.10×0.02
T/K	140.00(10)
Crystal System	monoclinic
Space Group	$P2_1/c$
a/Å	17.2179(6)
b/Å	8.7318(2)
c/Å	21.9955(7)
α/°	90
β/°	103.469(4)
γ/°	90
V/Å^3	3215.94(18)
Z	4
Z'	1
Wavelength/Å	1.54184
Radiation type	Cu$K\alpha$
Θ_{min}/°	4.133
Θ_{max}/°	72.735
Measured Refl's.	15995
Indep't Refl's	6239
Refl's I≥2σ(I)	4338
R_{int}	0.0742
Parameters	358
Restraints	0
Largest Peak/e Å^{-3}	0.449
Deepest Hole/e Å^{-3}	–0.642
GooF	1.016
wR_2 (all data)	0.1752
wR_2	0.1543
R_1 (all data)	0.0942
R_1	0.0621
CCDC number	2473519

Compound **3**

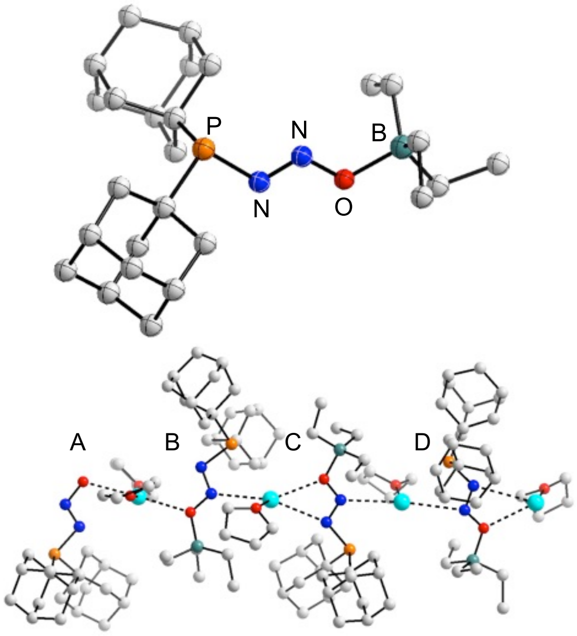

Figure S24. Molecular structure of compound **3** in the crystal (from left to right diazotates A to D). Thermal ellipsoids are at 40% probability and hydrogen atoms are not depicted.

Table S3. Selected bond lengths in Å.

	**A**	**B**	**C**	**D**
N–N	1.230(13)	1.318(19)	1.245(13)	1.245(16)
N–O	1.369(12)	1.294(16)	1.314(13)	1.295(15)

Structure Quality Indicators

**Reflections:**	d min (CuKα) 2Θ=79.9° 1.20	I/σ(I) 16.2	Rint m=7.43 8.71%	Full 79.9° 99.9
**Refinement:**	Shift 0.000	Max Peak 1.5	Min Peak -0.7	GooF 1.601

A colorless plate-shaped crystal with dimensions 0.20 × 0.15 × 0.01 mm^3 was mounted. Data were collected using a XtaLAB Synergy R, DW system, HyPix-Arc 150 diffractometer operating at T = 140.00(10) K. Data were measured using ω scans with CuK_α radiation. The diffraction pattern was indexed, and the total number of runs and images was based on the strategy calculation from the program CrysAlisPro[6] system (CCD 43.136a 64-bit (release 23-08-2024)). The maximum resolution achieved was Θ = 39.971° (1.20 Å). The unit cell was refined using CrysAlisPro 1.171.43.135a (Rigaku OD, 2024) on 19622 reflections, 33% of the observed reflections. Data reduction, scaling and absorption corrections were performed using CrysAlisPro 1.171.43.135a (Rigaku OD, 2024). The final completeness is 99.90 % out to 39.971° in Θ. A gaussian absorption correction was performed using CrysAlisPro 1.171.43.135a (Rigaku Oxford Diffraction, 2024) Numerical absorption correction based on gaussian integration over a multifaceted crystal model Empirical absorption correction using spherical harmonics, implemented in SCALE3 ABSPACK scaling algorithm. The absorption

coefficient μ of this material is 2.088 mm^{-1} at this wavelength (λ = 1.54184Å) and the minimum and maximum transmissions are 0.557 and 1.000. The structure was solved in the space group $P\bar{1}$ (# 2) by the ShelXT (Sheldrick, 2015) structure solution program using dual methods and refined by full matrix least squares minimisation on $\boldsymbol{F^2}$ using version 2019/3 of ShelXL 2019/3 (Sheldrick, 2015).[7] All non-hydrogen atoms were refined anisotropically. Hydrogen atom positions were calculated geometrically and refined using the riding model. A solvent mask was calculated, and 101 electrons were found in a volume of 433$Å^3$ in one void per unit cell. This is consistent with the presence of 1.25 THF per Asymmetric Unit which account for 100 electrons per unit cell. There is a single formula unit in the asymmetric unit, which is represented by the reported sum formula. In other words: Z is 2 and Z' is 1. The moiety formula is $C_{124}H_{218}B_4K_4N_8O_9P_4$, 1.25[$C_4H_8O$].

Table S4. Crystal data and structure refinement for **3**.

Compound	GA-199
Formula	$C_{129}H_{228}B_4K_4N_8O_{10.25}P_4$
$D_{calc.}$/ g cm^{-3}	1.186
μ/mm^{-1}	2.088
Formula Weight	2378.70
Colour	colorless
Shape	plate-shaped
Size/mm^3	0.20×0.15×0.01
T/K	140.00(10)
Crystal System	triclinic
Space Group	$P\bar{1}$
a/Å	16.8002(4)
b/Å	17.1154(7)
c/Å	24.4350(4)
α/°	96.603(3)
β/°	106.6979(18)
γ/°	92.909(3)
V/Å^3	6659.2(3)
Z	2
Z'	1
Wavelength/Å	1.54184
Radiation type	Cu$K\alpha$
Θ_{min}/°	1.905
Θ_{max}/°	39.971
Measured Refl's.	59870
Indep't Refl's	8056
Refl's I≥2σ(I)	5759
R_{int}	0.0871
Parameters	1390
Restraints	2306
Largest Peak/e Å^{-3}	1.481
Deepest Hole/e Å^{-3}	–0.719
GooF	1.601
wR_2 (all data)	0.3829
wR_2	0.3621
R_1 (all data)	0.1566
R_1	0.1262
CCDC number	2473517

Compound **5**

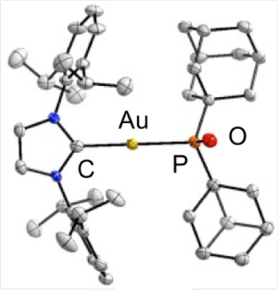

Figure S25. Molecular structure of compound **5** in the crystal. Thermal ellipsoids are at 40% probability and hydrogen atoms are not depicted. Selected bond lengths (Å) and angles (°): Au–P 2.2972(7), Au–C 2.041(3); C–Au–P 171.26(8).

Structure Quality Indicators

**Reflections:**	d min (CuKα) 2Θ=149.4° 0.80	I/σ(I) 32.3	Rint m=4.53 3.34%	Full 135.4° 96% to 149.4° 99.9
**Refinement:**	Shift 0.002	Max Peak 1.2	Min Peak -1.5	GooF 1.042

A colorless plate-shaped crystal with dimensions 0.15 × 0.05 × 0.01 mm^3 was mounted. Data were collected using a XtaLAB Synergy R, DW system, HyPix-Arc 150 diffractometer operating at T = 140.00(10) K. Data were measured using ω scans with CuK_α radiation. The diffraction pattern was indexed, and the total number of runs and images was based on the strategy calculation from the program CrysAlisPro[6] system (CCD 43.137a 64-bit (release 10-09-2024)). The maximum resolution achieved was Θ = 74.679° (0.80 Å). The unit cell was refined using CrysAlisPro 1.171.43.135a (Rigaku OD, 2024) on 24178 reflections, 51% of the observed reflections. Data reduction, scaling and absorption corrections were performed using CrysAlisPro 1.171.43.135a (Rigaku OD, 2024). The final completeness is 99.90 % out to 74.679° in Θ. A Gaussian absorption correction was performed using CrysAlisPro 1.171.43.135a (Rigaku Oxford Diffraction, 2024) Numerical absorption correction based on Gaussian integration over a multifaceted crystal model. Empirical absorption correction using spherical harmonics as implemented in SCALE3 ABSPACK scaling algorithm. The absorption coefficient μ of this material is 5.664 mm^{-1} at this wavelength (λ = 1.54184Å) and the minimum and maximum transmissions are 0.534 and 1.000. The structure was solved in the space group $P2_1/c$ (# 14) by the ShelXT 2018/2 (Sheldrick, 2015) structure solution program using dual methods and refined by full matrix least squares minimisation on $\boldsymbol{F}^2$ using version 2019/3 of ShelXL 2019/3 (Sheldrick, 2015).[7] All non-hydrogen atoms were refined anisotropically. Hydrogen atom positions were calculated geometrically and refined using the riding model. Hydrogen atom positions were calculated geometrically and refined using the riding model. A solvent mask was calculated, and 433 electrons were found in a volume of 1773$Å^3$ in 1 void per unit cell. This is consistent with the presence of 2.5 THF per Asymmetric Unit which account for 400 electrons per unit cell. There is a single formula unit in the asymmetric unit, which is represented by the reported sum formula. In other words: Z is 4 and Z' is 1.

The moiety formula is $C_{47}H_{66}AuN_2OP$, 2.5[C_4H_8O].

Table S5. Crystal data and structure refinement for **5**.

Compound	ga-210
Formula	$C_{57}H_{86}AuN_2O_{3.5}P$
$D_{calc.}$/ g cm^{-3}	1.317
μ/mm^{-1}	5.664
Formula Weight	1083.21
Colour	colorless
Shape	plate-shaped
Size/mm^3	0.15×0.05×0.01
T/K	140.00(10)
Crystal System	monoclinic
Space Group	$P2_1/c$
a/Å	17.92474(18)
b/Å	22.02986(18)
c/Å	15.23246(18)
α/°	90
β/°	114.7071(13)
γ/°	90
V/Å^3	5464.35(11)
Z	4
Z'	1
Wavelength/Å	1.54184
Radiation type	Cu$K\alpha$
Θ_{min}/°	3.375
Θ_{max}/°	74.679
Measured Refl's.	47812
Indep't Refl's	10776
Refl's I≥2σ(I)	9137
R_{int}	0.0334
Parameters	478
Restraints	12
Largest Peak/e Å^{-3}	1.152
Deepest Hole/e Å^{-3}	–1.511
GooF	1.042
wR_2 (all data)	0.0717
wR_2	0.0697
R_1 (all data)	0.0338
R_1	0.0274
CCDC number	2473518

Compound **6:**

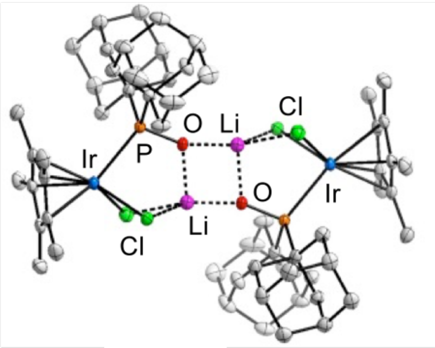

Figure S26. Molecular structure of compound **6** in the crystal. Thermal ellipsoids are at 40% probability and hydrogen atoms are not depicted. Selected bond lengths (Å) and angles (°): Ir–P 2.3841(9), Ir–Cl1 2.4413(9); Ir–Cl2 2.4270(8).

Structure Quality Indicators

**Reflections:**	d min (CuKα) 2Θ=151.2° 0.80	I/σ(I) 54.1	Rint m=7.07 2.62%	Full 135.4° 98% to 151.2° 99.8
**Refinement:**	Shift 0.001	Max Peak 1.4	Min Peak -1.0	GooF 1.056

A clear intense orange prism-shaped crystal with dimensions 0.09 × 0.07 × 0.04 mm[3] was mounted. Data were collected using a XtaLAB Synergy R, DW system, HyPix-Arc 150 diffractometer operating at T = 139.99(10) K. Data were measured using ω scans with Cu K_α radiation. The diffraction pattern was indexed, and the total number of runs and images was based on the strategy calculation from the program CrysAlisPro[6] system (CCD 43.130a 64-bit (release 05-07-2024)). The maximum resolution achieved was Θ = 75.625° (0.80 Å). The unit cell was refined using CrysAlisPro 1.171.43.130a (Rigaku OD, 2024) on 29587 reflections, 65% of the observed reflections. Data reduction, scaling and absorption corrections were performed using CrysAlisPro 1.171.43.130a (Rigaku OD, 2024). The final completeness is 99.80 % out to 75.625° in Θ. A Gaussian absorption correction was performed using CrysAlisPro 1.171.43.130a (Rigaku Oxford Diffraction, 2024) Numerical absorption correction based on Gaussian integration over a multifaceted crystal model. Empirical absorption correction using spherical harmonics as implemented in SCALE3 ABSPACK scaling algorithm. The absorption coefficient μ of this material is 10.118 mm^{-1} at this wavelength (λ = 1.54184Å) and the minimum and maximum transmissions are 0.473 and 0.759. The structure was solved in the space group $P2_1/c$ (# 14) by the ShelXT 2018/2 (Sheldrick, 2015) structure solution program using dual methods and refined by full matrix least squares minimisation on $\boldsymbol{F^2}$ using version 2019/3 of ShelXL 2019/3 (Sheldrick, 2015).[7] All non-hydrogen atoms were refined anisotropically. Hydrogen atom positions were calculated geometrically and refined using the riding model. The value of Z' is 0.5. This means that only half of the formula unit is present in the asymmetric unit, with the other half consisting of symmetry equivalent atoms. The moiety formula is $C_{60}H_{90}Cl_4Ir_2Li_2O_2P_2$, C_4H_8O.

Table S6. Crystal data and structure refinement for **6**.

Compound	ga-189-ir-1
Formula	$C_{64}H_{98}Cl_4Ir_2Li_2O_3P_2$
$D_{calc.}$/ g cm^{-3}	1.552
μ/mm^{-1}	10.118
Formula Weight	1517.44
Colour	clear intense orange
Shape	prism-shaped
Size/mm^3	0.09×0.07×0.04
T/K	139.99(10)
Crystal System	monoclinic
Space Group	$P2_1/c$
a/Å	10.52239(8)
b/Å	18.32736(16)
c/Å	17.04898(11)
α/°	90
β/°	99.0460(6)
γ/°	90
V/Å^3	3246.96(4)
Z	2
Z'	0.5
Wavelength/Å	1.54184
Radiation type	Cu$K\alpha$
Θ_{min}/°	3.565
Θ_{max}/°	75.625
Measured Refl's.	45359
Indep't Refl's	6598
Refl's I≥2σ(I)	6040
R_{int}	0.0262
Parameters	457
Restraints	417
Largest Peak/e Å^{-3}	1.448
Deepest Hole/e Å^{-3}	–1.008
GooF	1.056
wR_2 (all data)	0.0779
wR_2	0.0765
R_1 (all data)	0.0305
R_1	0.0277
CCDC number	2473515

6. Computational details

General:

All calculations were performed using the Gaussian 16 package (revision C.01).[8] The geometry optimizations and vibrational frequencies were calculated by using M062X[9] level of theory. The Def2-TZVPP basis set were employed for C, H, N, O, and P atoms.[10,11] Frequency calculations at the same level of theory were performed to identify the number of imaginary frequencies (Number of imaginary frequencies (NIMAG): 0 for local minimum, 1 for transition states), and provide the thermal corrections of Gibbs free energy. The corrections of Gibbs free energy from frequency calculations were added to the absolute energy to obtain the Gibbs free energy. The NBO analysis[12] was performed with the Version 3.1 of the NBO program which was implemented in the G16 C.01 version of the Gaussian program.[13] Frontier orbitals were visualized in Avogadro[14] and NBO were visualized in Chemcraft.[15] All calculations were performed in gas-phase.

Compound **1**$^{-}$ and **2**$^{-}$ mean compound **1** and **2** without cation.

Table S7. Calculated absolute energy, E(SCF), and free energies at 298 K, G^{298}, for compound of interest (calculated at the M062X/Def2-TZVPP level).

Compound	**1**$^{-}$	**1**$_{TS}^{-}$	**1**$_{cis}^{-}$
E(SCF) (a.u.)	–1306.237711	–1306.211057	–1306.242090
NIMAG	0	1	0
G^{298} (a.u.)	–1305.802907	–1305.776704	–1305.804398
ΔG_{corr} (a.u.)	0.434815	0.434353	0.437692
ΔG (a.u.)	–1305.802896	–1305.776704	–1305.804398
$\Delta\Delta G$ (kcal/mol)	--	+16.434	–0.943

Compound	**2**$^{-}$	**2**$_{TS}^{-}$	**2**$_{trans}^{-}$
E(SCF) (a.u.)	–841.713025	–841.682200	–841.709148
NIMAG	0	1	0
G^{298} (a.u.)	–841.494177	–841.466466	–841.493012
ΔG_{corr} (a.u.)	0.218843	0.215735	0.216144
ΔG (a.u.)	–841.494182	–841.466465	–841.493004
$\Delta\Delta G$ (kcal/mol)	--	+17.397	+0.739

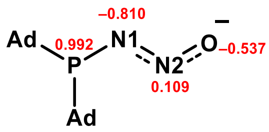

Figure S27. Calculated NPA charges of compound **1**⁻ on selected atoms (calculated at the M062X/Def2-TZVPP level).

Table S8. Comparison of the structural parameters of compound **1**⁻; experimental data from XRD and computed data (calculated at the M062X/Def2-TZVPP level).

	**1**⁻ (exp)	**1**⁻ (calc)
P–N1 (Å)	1.723	1.703
WBI	--	0.93
N1–N2 (Å)	1.257	1.307
WBI	--	1.48
N2–O (Å)	1.259	1.236
WBI	--	1.56
P-N1-N2 (°)	110.6	111.2
N1-N2-O (°)	115.4	115.0

Table S9. NBO analysis of the selected bonds and lone pair of compound **1**⁻ (calculated at the M062X/Def2-TZVPP level).

	Occupation Number		%	%s	%p	%d
P–N1	1.96	P	31.32	17.93	81.65	0.38
		N1	68.68	26.10	73.20	0.69
N1–N2	1.99	N1	49.49	28.63	70.76	0.57
		N2	50.51	29.67	70.18	0.14
N2–O (π)	1.99	N2	31.69	0.03	99.25	0.68
		O	68.31	0.04	99.86	0.10
N2–O (σ)	1.99	N2	45.41	27.03	72.77	0.18
		O	54.59	27.54	72.21	0.22
P	1.93		--	49.38	50.60	0.01
N1	1.96		--	45.45	54.28	0.27
N1	1.46		--	0.00	99.46	0.52
N2	1.97		--	45.07	54.83	0.10
O	1.99		--	71.41	28.58	0.01
O	1.94		--	1.78	98.17	0.04

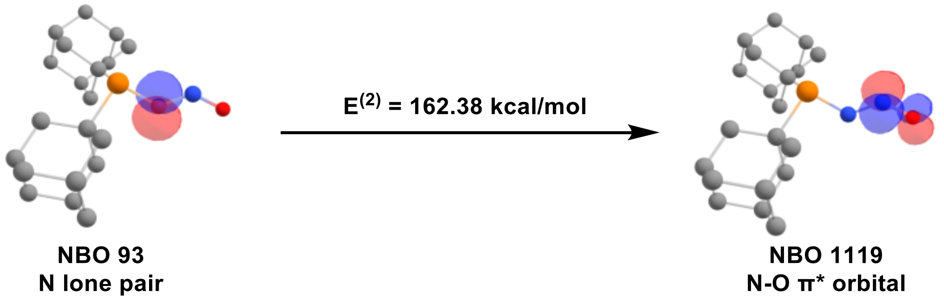

Figure S28. Selected NBO of compound **1**⁻ from second-order perturbation theory analysis (calculated at the M062X/Def2-TZVPP level).

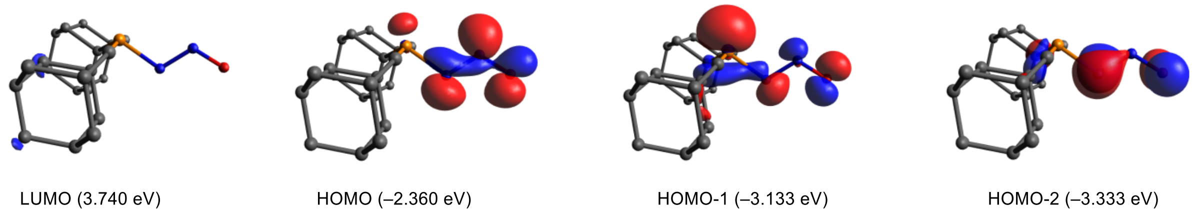

Figure S29. Selected surface diagrams of the frontier Kohn-Sham orbitals of complex **1**⁻ (M062X/Def2-TZVPP), at an isodensity value of 0.06. Color code: deep-blue, nitrogen; red, oxygen; gray, carbon; orange, phosphorus. Hydrogen atoms are omitted.

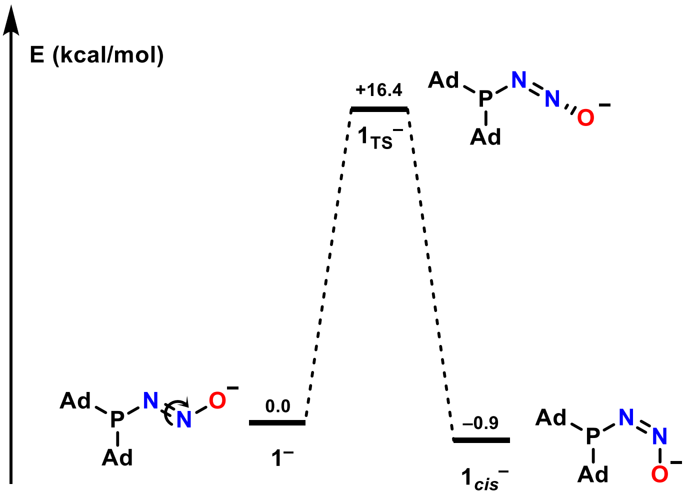

Figure S30. Energy plot for NNO *cis-trans* isomerization (calculated at the M062X/Def2-TZVPP level).

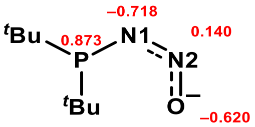

Figure S31. Calculated NPA charges of compound **2**⁻ on selected atoms (calculated at the M062X/Def2-TZVPP level).

Table S10. Comparison of the structural parameters of compound **2**⁻; experimental data from XRD and computed data (calculated at the M062X/Def2-TZVPP level).

	**2**⁻ (exp)	**2**⁻ (calc)
P–N1 (Å)	1.775	1.754
WBI	--	0.87
N1–N2 (Å)	1.274	1.269
WBI	--	1.63
N2–O (Å)	1.278	1.264
WBI	--	1.41
P-N1-N2 (°)	118.3	116.4
N1-N2-O (°)	118.4	118.3

Table S11. NBO analysis of the selected bonds and lone pair of compound **2**⁻ (calculated at the M062X/Def2-TZVPP level).

	Occupation Number		%	%s	%p	%d
P–N1	1.97	P	33.16	17.14	82.40	0.43
		N1	66.84	23.50	75.97	0.51
N1–N2 (σ)	1.99	N1	49.41	32.74	66.66	0.56
		N2	50.59	33.32	66.47	0.20
N1–N2 (π)	1.97	N1	64.37	0.03	99.61	0.34
		N2	35.63	0.12	99.52	0.35
N2–O (σ)	1.99	N2	45.37	26.42	73.31	0.25
		O	54.63	24.32	75.50	0.16
P	1.94		--	51.94	48.04	0.01
N1	1.93		--	43.69	56.10	0.20
N2	1.92		--	41.17	58.70	0.12
O	1.97		--	71.61	28.38	0.01
O	1.92		--	4.28	95.69	0.03
O	1.60		--	0.01	99.93	0.07

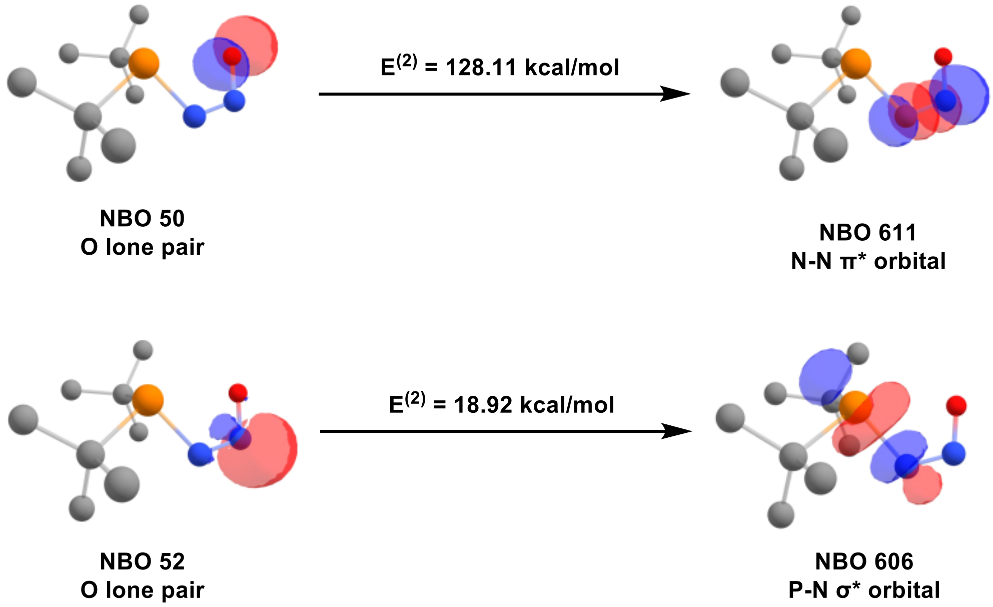

Figure S32. Selected NBO of compound **2**⁻ from second-order perturbation theory analysis (calculated at the M062X/Def2-TZVPP level).

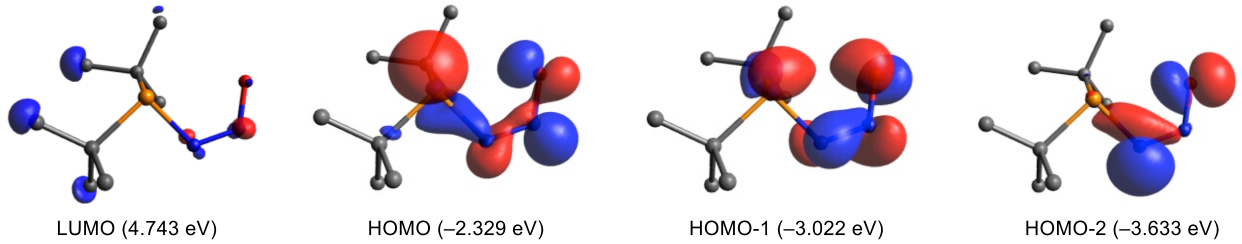

Figure S33. Selected surface diagrams of the frontier Kohn-Sham orbitals of complex **1**⁻ (M062X/Def2-TZVPP), at an isodensity value of 0.06. Color code: deep-blue, nitrogen; red, oxygen; gray, carbon; orange, phosphorus. Hydrogen atoms are omitted.

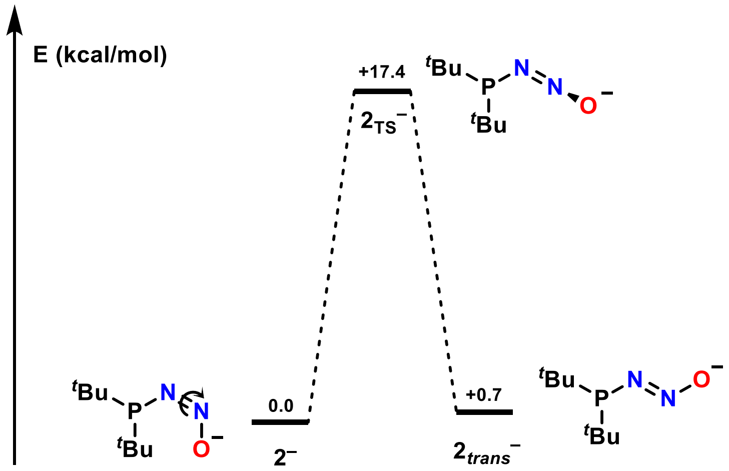

Figure S34. Energy plot for NNO *cis-trans* isomerization (calculated at the M062X/Def2-TZVPP level).

Compound **1**⁻ (–1 0, M062X/Def2-TZVPP)

P 0.0032196092 1.0192374998 -0.8604409024
O -0.0683679141 4.3966745079 0.5163137295
N 0.0022325626 2.2644106165 0.3008024987
N -0.0767114601 3.4393323685 -0.2652109827
C -1.5263174162 0.0623503538 -0.2761140435
C 3.1724250262 -2.2692888155 0.4900534344
H 4.1259489828 -2.7487153862 0.7365299103
H 2.3926437587 -3.0339056899 0.5644019897
C -4.2877458403 0.2019227824 0.8331555572
H -4.3198914295 1.2422564084 1.1668355538
H -5.2554343308 -0.2536643344 1.0711051161
C -1.8124675573 0.1234292736 1.2346742357
H -1.0257799240 -0.3819058043 1.7953260523
H -1.8016535195 1.1667755081 1.5530478239
C -4.0363169843 0.1487283675 -0.6763998111
H -4.8292845382 0.6889178578 -1.2013495761
C 1.5372553926 0.0664578574 -0.2952411463
C -2.8912904161 -2.0595082431 -0.4158641630
H -2.8738139736 -3.1028723976 -0.7447029200
C 1.5241765239 -0.5002749467 1.1308086350
H 0.7536133911 -1.2698658800 1.2217749962
H 1.2766837991 0.3032079378 1.8295993308
C 2.6737491318 1.1090052428 -0.3813667844
H 2.4540406817 1.9273285132 0.3064161905
H 2.6943622775 1.5390155578 -1.3887274420
C 2.8810613677 -1.1325580049 1.4773210519
H 2.8458440636 -1.5327606249 2.4948239564
C 4.3287077969 -0.6588455373 -1.0307832151
H 4.3820294282 -0.2649988858 -2.0499171919
H 5.2989943465 -1.1129947521 -0.8018139628
C 3.9880933775 -0.0790528338 1.3779327614
H 4.9548283119 -0.5230716791 1.6399129876
H 3.7929658543 0.7314865235 2.0848698122
C -3.1602102510 -0.5390135648 1.5571890324
H -3.3317567064 -0.4994366422 2.6367287093
C 3.2199613535 -1.7116098634 -0.9378633434
H 3.4249451568 -2.5242258465 -1.6411578981
C -1.5381589363 -1.4034289585 -0.7311248044
H -1.3310149311 -1.4622333630 -1.8058690721
H -0.7517489834 -1.9630252095 -0.2182533763
C -2.6837472949 0.7939743400 -0.9948723025
H -2.5085601003 0.7731249168 -2.0745134333
H -2.6790271877 1.8451742014 -0.6918364183
C 4.0297085082 0.4781810294 -0.0486892344
H 4.8100184343 1.2407857085 -0.1211479748
C -4.0159858584 -1.3119067073 -1.1394823522
H -3.8566307568 -1.3564484419 -2.2210384323
H -4.9786503404 -1.7901909273 -0.9288510957
C -3.1385554095 -2.0013298552 1.0965104323
H -4.0883421843 -2.4901967939 1.3394382893
H -2.3467789011 -2.5442350686 1.6221992621
C 1.8695082513 -1.0667090240 -1.2831490111
H 1.0914391207 -1.8330232608 -1.2676312599
H 1.8969070786 -0.6615989786 -2.2994063595

Compound $\mathbf{1}_{TS}^{-}$ (–1 0, M062X/Def2-TZVPP)

P -0.0121707101 1.0946088975 -0.7942801621
O 1.2630433888 4.0377949445 0.2824228455
N -0.0593211476 2.3230704869 0.2820195839
N 0.1317658477 3.6111073756 0.0739321188
C -1.5740684942 0.1528031172 -0.2630371645
C 3.0063925550 -2.4082461201 0.4347203408
H 3.9362973642 -2.9417795839 0.6599537561
H 2.1887838750 -3.1334783669 0.5002478444
C -4.3379409867 0.3527357669 0.8324089410
H -4.3441595756 1.3900141946 1.1775738082
H -5.3188671732 -0.0792746972 1.0605335948
C -1.8671940482 0.2000832466 1.2455938391
H -1.0975911339 -0.3352611464 1.8029955499
H -1.8306539069 1.2414783539 1.5708619343
C -4.0794164517 0.3103580992 -0.6765501239
H -4.8551781051 0.8776187831 -1.1991557450
C 1.4837096761 0.0190767391 -0.2790416582
C -2.9947738661 -1.9294808621 -0.4361273854
H -3.0027893133 -2.9691195826 -0.7770922853
C 1.4575361903 -0.5748637967 1.1332937358
H 0.6496866810 -1.3057959541 1.2238761525
H 1.2621536249 0.2279885134 1.8510889685
C 2.6682751535 1.0039515599 -0.3582570796
H 2.4921786472 1.8327721821 0.3308260609
H 2.7031587479 1.4445000633 -1.3611743860
C 2.7841193364 -1.2818085153 1.4523759347
H 2.7405167908 -1.7050879607 2.4606493219
C 4.2295547416 -0.8250158645 -1.0606845109
H 4.2945954836 -0.4106033870 -2.0710489524
H 5.1771645607 -1.3340109790 -0.8513642221
C 3.9410573555 -0.2826152380 1.3632960691
H 4.8870962222 -0.7798482772 1.6057680028
H 3.7933034853 0.5214888429 2.0889610929
C -3.2348904586 -0.4264076305 1.5544283361
H -3.4122980808 -0.3958274850 2.6335390937
C 3.0690906454 -1.8214548649 -0.9815892825
H 3.2267499563 -2.6283114737 -1.7040088890
C -1.6218317009 -1.3063617027 -0.7354531935
H -1.4098192654 -1.3591517648 -1.8095438268
H -0.8560848003 -1.8943858243 -0.2243751570
C -2.7078470833 0.9221532045 -0.9778250546
H -2.5262899075 0.9098548563 -2.0569319862
H -2.6722540845 1.9669480002 -0.6566072764
C 3.9951965655 0.3034142089 -0.0512132264
H 4.8106919817 1.0292977776 -0.1150267047
C -4.0955849480 -1.1447994930 -1.1568407087
H -3.9309251413 -1.1803450352 -2.2379896611
H -5.0717845078 -1.6002726245 -0.9576054474
C -3.2503358290 -1.8833537919 1.0755567664
H -4.2148515846 -2.3488326855 1.3063325662
H -2.4770776548 -2.4539153832 1.5994433668
C 1.7469402474 -1.1030801514 -1.2968377883
H 0.9374289471 -1.8357138464 -1.2931226944
H 1.7839869648 -0.6742110230 -2.3037468826

Compound **1**$_{cis}^{-}$ (–1 0, M062X/Def2-TZVPP)

```
P 0.0279184600 1.1695510637 -0.7799287175
O -1.6090877855 3.2461935091 -0.2280218267
N 0.2237417382 2.3391007145 0.5148843880
N -0.6183149870 3.2872831563 0.5555057641
C -1.4857759702 0.1141429417 -0.2826654675
C 3.2752740140 -2.0702639339 0.5381128841
H 4.2363985851 -2.5439154773 0.7663897133
H 2.4959801187 -2.8259641353 0.6794653029
C -4.3232047509 0.0669068421 0.5725812119
H -4.4837640851 1.1376431068 0.7214332625
H -5.2593283659 -0.4500858567 0.8127020908
C -1.9116332601 0.3530065479 1.1750738110
H -1.1130990741 0.0424909794 1.8540484661
H -2.0682788473 1.4208706128 1.3275836879
C -3.9337076080 -0.1976896389 -0.8860865021
H -4.7315536307 0.1543691008 -1.5464905245
C 1.5979210192 0.2227134543 -0.2813617632
C -2.5856276062 -2.1698189946 -0.1789194857
H -2.4082995088 -3.2389455806 -0.3318702201
C 1.6609460353 -0.2622274731 1.1762289520
H 0.8841299647 -1.0057035810 1.3692306856
H 1.4678293803 0.5899378434 1.8314439843
C 2.7362943483 1.2546199565 -0.4786461502
H 2.5478107084 2.1109535813 0.1684673606
H 2.7149255693 1.6165498615 -1.5122715026
C 3.0297863364 -0.8882467252 1.4837414002
H 3.0436520651 -1.2404355573 2.5194632146
C 4.3711063904 -0.5386626304 -1.1049507135
H 4.3841393318 -0.1958010552 -2.1437949591
H 5.3495609330 -0.9834722803 -0.8919766890
C 4.1309889247 0.1571607571 1.2831829949
H 5.1097086116 -0.2741486243 1.5216852159
H 3.9663720925 1.0009479713 1.9584193252
C -3.1998095076 -0.4171403289 1.4959061418
H -3.4787328608 -0.2338997869 2.5376197344
C 3.2651031010 -1.5793031257 -0.9150969386
H 3.4369441494 -2.4254964821 -1.5871095532
C -1.2963709058 -1.3976722090 -0.5035258072
H -1.0110879326 -1.5859226616 -1.5440106149
H -0.4978239762 -1.7862857635 0.1294638396
C -2.6388527472 0.5540603642 -1.2157644797
H -2.3506802231 0.3515640447 -2.2523300618
H -2.7790383356 1.6291941540 -1.1178221553
C 4.1078194616 0.6438926298 -0.1693979938
H 4.8801229154 1.4052456821 -0.3123911377
C -3.7213110269 -1.6997419586 -1.0909025774
H -3.4674321916 -1.9004796809 -2.1362717790
H -4.6392098901 -2.2529838708 -0.8625190237
C -2.9733175295 -1.9172630176 1.2821058335
H -3.8803682017 -2.4793378976 1.5304016006
H -2.1766819309 -2.2703044917 1.9447097513
C 1.8984918796 -0.9525528899 -1.2307527468
H 1.1383509924 -1.7281010679 -1.1519994410
H 1.8806682070 -0.5915076264 -2.2644708845
```

Compound **2**$^{-}$ (–1 0, M062X/Def2-TZVPP)

```
P 0.0076939640 -0.3181170886 -0.6640134660
O -2.2564877225 -1.7568777615 -0.4407745922
N -0.2551712962 -1.6816424682 0.4073994153
N -1.3754649329 -2.2692556678 0.3069053746
C 1.7856844436 -0.0488857004 -0.0306625355
C -1.0701930897 1.0979507098 0.0407005018
C 1.9252027342 0.1143200288 1.4822187934
H 1.5069278235 1.0574021040 1.8354451250
H 2.9848214981 0.0982367877 1.7667000578
H 1.4135697719 -0.7036274284 1.9897570196
C 2.4864740111 1.1006365593 -0.7608209593
H 2.3046078063 1.0454669936 -1.8370642855
H 3.5680888722 1.0308475578 -0.5983981751
H 2.1690145341 2.0812899178 -0.4138352457
C 2.5156572609 -1.3391743674 -0.4364593166
H 2.0767300349 -2.2014983522 0.0612462901
H 3.5734896019 -1.2602382310 -0.1610847755
H 2.4547720258 -1.4973648558 -1.5160581783
C -0.3464869847 2.4451619455 0.0493917249
H 0.4521937109 2.4893395427 0.7881727639
H -1.0673185356 3.2330437148 0.2960366462
H 0.0750452531 2.6789133087 -0.9308750450
C -2.2649166634 1.2485293068 -0.9113539571
H -1.9271434917 1.5485296665 -1.9059546753
H -2.9353990721 2.0282918203 -0.5307065463
H -2.8083289715 0.3108895127 -0.9965984310
C -1.5855439983 0.7837698508 1.4434458946
H -2.1838853080 -0.1261024504 1.4301142909
H -2.2122288013 1.6118495190 1.7978281027
H -0.7692771211 0.6439462513 2.1527793880
```

Compound $\mathbf{2}_{\mathbf{TS}}^{-}$ (–1 0, M062X/Def2-TZVPP)

```
P 0.0705221786 -0.2179934323 -0.6496840828
O 2.9852467131 -1.7906771406 0.0460752295
N 0.8882326914 -1.2978418280 0.2628146212
N 1.8219325849 -2.1535099859 -0.1020952976
C 0.6067599899 1.5040450566 0.0107145036
C -1.6955758734 -0.5147143613 0.0058525430
C 0.3763586047 1.7228140055 1.5005672934
H -0.6771163541 1.8951497551 1.7281083441
H 0.9353588058 2.5993467952 1.8513973201
H 0.7200219786 0.8510779690 2.0624456634
C -0.0195647060 2.6311569760 -0.8108510289
H 0.0823470540 2.4300791250 -1.8805123326
H 0.4902717311 3.5783478297 -0.5979983129
H -1.0775841713 2.7745835684 -0.5954196694
C 2.1160782921 1.5150552022 -0.2499768318
H 2.6091495966 0.7001031082 0.2814724681
H 2.5382904083 2.4748143162 0.0695712885
H 2.3292013065 1.3846161002 -1.3144039527
C -2.6434157947 0.6345613888 -0.3240285463
H -2.4757981413 1.4940691772 0.3258456471
H -3.6827074120 0.3151024507 -0.1851147152
H -2.5285921194 0.9618302713 -1.3611587805
```

C -2.1475826826 -1.7580788720 -0.7700062638
H -2.1807904627 -1.5613894718 -1.8435382765
H -3.1468022976 -2.0654367000 -0.4411495227
H -1.4528579410 -2.5819608050 -0.5967421521
C -1.7648653898 -0.8477406494 1.4951308813
H -1.0171425507 -1.6031285781 1.7384256962
H -2.7592191410 -1.2388300654 1.7434414283
H -1.5861482551 0.0238140335 2.1225387256

Compound $\mathbf{2}_{trans}^{-}$ (–1 0, M062X/Def2-TZVPP)

P -0.1338239893 -0.0373008002 -0.6890984078
O -3.6111764226 -0.6438495548 0.2360171519
N -1.4962227379 -0.2944365318 0.2994898694
N -2.5797595760 -0.4379187032 -0.4145747106
C 1.0124174533 -1.3749482178 0.0165898617
C 0.4419663844 1.6346424514 0.0138690595
C 1.3255641783 -1.2631574846 1.5056127705
H 2.0211503569 -0.4477623613 1.7105656380
H 1.7928989440 -2.1896165670 1.8613757818
H 0.4087187467 -1.0959671260 2.0728257443
C 2.3077139971 -1.4447697584 -0.7942145971
H 2.0918462623 -1.5418362310 -1.8602938790
H 2.8943304492 -2.3174667648 -0.4846752831
H 2.9321178083 -0.5620322813 -0.6579937472
C 0.2374454160 -2.6777017046 -0.2151535562
H -0.6705832381 -2.6958627436 0.3864440380
H 0.8676408928 -3.5326772489 0.0537775814
H -0.0556242321 -2.7806576814 -1.2629482480
C 1.9063788426 1.9150288949 -0.3119116460
H 2.5775086943 1.3278667871 0.3168701884
H 2.1333713111 2.9730461463 -0.1374533163
H 2.1325985960 1.6901330636 -1.3579002025
C -0.4303747704 2.6373977851 -0.7542209341
H -0.2433399305 2.5814756924 -1.8281850518
H -0.2160996542 3.6565737362 -0.4140321277
H -1.4892523840 2.4307444383 -0.5863945786
C 0.1826050986 1.8439860239 1.5061840967
H -0.8331793860 1.5400485487 1.7577769764
H 0.3126455492 2.9042750912 1.7550448663
H 0.8695990231 1.2736426071 2.1285980692

7. References

1) S. Kundu, S. Sinhababu, M. M. Siddiqui, A. V. Luebben, B. Dittrich, T. Yang, G. Frenking and H. W. Roesky, *J. Am. Chem. Soc.*, 2018, **140**, 9409–9412.
2) J. E. Borger, A. W. Ehlers, M. Lutz, J.C. Slootweg, K. Lammertsma, *Angew. Chem. Int. Ed.,* 2016, **55**, 613–617.
3) H. Schneider, D. Schmidt, U. Radius, *Chem. Commun.,* 2015, **51**,10138−10141.
4) S. Zhang, H. Neumann and M. Beller, *Chem. Eur. J.*, 2018, **24**, 67−70.
5) J. R. Goerlich, R. Schmutzler, *Phosphorus Sulfur Silicon Relat. Elem.,* 1993, **81**, 141–14.
6) *CrysAlis^{Pro}* Software System, Rigaku Oxford Diffraction (2024-25).
7) G. M. Sheldrick, *Acta Crystallogr., Sect. A,* 2015, **71**, 3–8.
8) M. J. Frisch, G. W. Trucks, H. B. Schlegel, G. E. Scuseria, M. A. Robb, J. R. Cheeseman *et al.*, *Gaussian 16*, Gaussian, Inc., Wallingford CT, 2019.
9) Y. Zhao, N. E. Schultz and D. G. Truhlar, *J. Chem. Theory Comput.*, 2006, **2**, 364–382.
10) F. Weigend and R. Ahlrichs, *Phys. Chem. Chem. Phys.*, 2005, **7**, 3297.
11) B. P. Pritchard, D. Altarawy, B. Didier, T. D. Gibson and T. L. Windus, *J. Chem. Inf. Model.*, 2019, **59**, 4814–4820.
12) A. E. Reed, L. A. Curtiss and F. Weinhold, *Chem. Rev.*, 1988, **88**, 899–926.
13) E. D. Glendening, A. E. Reed, J. E. Carpenter and F. Weinhold, NBO Version 3.1.
14) M. D. Hanwell, D. E. Curtis, D. C. Lonie, T. Vandermeersch, E. Zurek and G. R. Hutchison, *J. Cheminform.*, 2012, **4**, 17.
15) Chemcraft, graphical software for visualization of quantum chemistry computations, Version 1.8, build 682, https://www.chemcraftprog.com
